# The Advantaged Salt Inducible *Suaeda salsa SsNRT2.5* and Its Promoter Significantly Enhance Nitrate Transport Efficiency and Salt Tolerance in Transgenic *Arabidopsis* and Rice

**DOI:** 10.1111/pbi.70600

**Published:** 2026-03-10

**Authors:** Ranran Liu, Chenyang Li, Runtai Zhao, Congcong Song, Yan Zhou, Ruxin Zhao, Na Sui, Lei Wang, Jie Song

**Affiliations:** ^1^ College of Life Science Shandong Normal University Jinan China; ^2^ College of Agriculture and Biology Liaocheng University Liaocheng China; ^3^ Key Laboratory of Ecological Safety and Sustainable Development in Arid Lands Xinjiang Institute of Ecology and Geography, Chinese Academy of Sciences Urumqi China

**Keywords:** low nitrogen, nitrate transport, salt tolerance, *SsNRT2.5*, *Suaeda salsa*

## Abstract

Efficient nitrogen (N) uptake is critical for crop yield, but soil salinization inhibits plant nitrogen acquisition. In this study, the nitrate (NO_3_^−^) transporter gene *SsNRT2.5* and its promoter from the halophyte *Suaeda salsa* was investigated to elucidate the functional role in NO_3_^−^ transport under salinity and low NO_3_^−^–N conditions. *SsNRT2.5* and its promoter were cloned and transformed into *Arabidopsis thaliana* and rice ( *Oryza sativa* L.) for functional identification, which included analyses of expression patterns, promoter *cis‐*element characterisation and phenotypic assessments under salt and low NO_3_^−^–N conditions. *SsNRT2.5* expression was significantly upregulated under salt stress and low NO_3_^−^–N conditions in *S. salsa* , which improved NO_3_^−^ transport in trangenic *Arabidopsis thaliana* and rice. Its promoter contained salt‐responsive (e.g., GT‐1, DRE) and N‐related (e.g., GATABOX) elements, which drove stronger salt‐induced *NRT2.5* expression than *AtNRT2.5* promoter. Transgenic *Arabidopsis* and rice with *SsNRT2.5* and its promoter showed enhanced NO_3_^−^ accumulation, reduced Na^+^ toxicity, and higher salt tolerance, as well as improved seed NO_3_^−^ storage and viability compared to WT. *SsNRT2.5* plays a key role in the adaptation of *S. salsa* to high saline and nitrogen‐limited environments, offering valuable genetic resources and theoretical insights for breeding salt‐tolerant crops and developing sustainable saline agriculture.

## 1 Introduction

The efficient N uptake and utilisation by plants are a fundamental determinant of crop yield. Soil N deficiency severely constrains agricultural output, compelling the annual application of large quantities of N fertilisers to sustain optimal yields. However, excessive N fertilisation reduces N use efficiency and causes significant environmental damage. Based on the dual requirements of ecological environmental protection and economic benefits, it is crucial to explore valuable genes that enhance plant growth under low N conditions to improve modern agricultural systems and develop sustainable agriculture (Zhang et al. 2013; Wu et al. 2020). Shi et al. (2022) systematically analysed genes associated with N uptake and metabolism in the high nitrogen‐use efficiency cultivar Kenong 9204 (KN9204) compared to the low nitrogen‐use efficiency cultivar Jing 411 in wheat. Physiological and yield data revealed that KN9204's N efficiency resulted from multiple factors, including elevated expression of root morphology and NO_3_^−^ uptake‐related genes (e.g., NPF and NRT2 families) as well as enhanced NO_3_^−^ uptake and transport efficiency during the reproductive growth stage (Shi et al. 2022). Therefore, the study of crop NO_3_^−^ uptake and utilisation patterns, and the selection and breeding of N‐efficient varieties have important scientific value from the perspectives of ecological environmental protection and economic benefits.

The NRT1/PTR (NPF, nitrate transporter 1/peptide transporter), NRT2, CLC (chloride channel), and SLAC1/SLAH (slow anion channel‐associated) have been identified as being associated with NO_3_^−^ uptake and transport (Orsel et al. 2004; Wang et al. 2012; Krapp et al. 2014). To adapt to fluctuating N availability in the environment, plants have evolved two distinct NO_3_^−^ uptake systems: a high‐affinity transport system (HATS) that operates under low NO_3_^−^ concentrations and a low‐affinity transport system (LATS) that functions when NO_3_^−^ is abundant. Under high NO_3_^−^ conditions, the low‐affinity NRT1 family plays a dominant role in uptake, whereas the high‐affinity NRT2 family predominates under low N availability (Orsel et al. 2004). *NRT2.5* belongs to the NRT2 family, which widely exists across all organisms and is initially characterised as the transporter for N limitation (Lezhneva et al. 2014). *NRT2.5* was expressed in both roots and shoots, with higher expression levels in the roots, and its transcription was suppressed by NO_3_^−^ supply (Lezhneva et al. 2014; Kotur and Glass 2015). The multiple functions of *NRT2.5* in different species, including coping with N starvation, participation in NO_3_^−^ transport in the phloem of buds, seed NO_3_^−^ accumulation, and NO_3_^−^ signal transduction guidance, etc. (Liu et al. 2020). In *Arabidopsis*, seven NRT2 family members (NRT2.1–2.7) have been identified (Orsel et al. 2004). Among these, NRT2.1, NRT2.2, and NRT2.4 exhibit high sequence conservation (> 87% amino acid identity) and localise to the root plasma membrane (Orsel et al. 2004; Chopin et al. 2007), whereas NRT2.5 has only 65% amino acid similarity to NRT2.1, NRT2.2, and NRT2.4. Functional complementation studies demonstrated that overexpression of *AtNRT2.5* in *Arabidopsis* mutant *nrt2.1‐1* partially restored the NO_3_^−^ uptake capacity, confirming its role as a high‐affinity NO_3_^−^ transporter (Lezhneva et al. 2014). These findings demonstrate that AtNRT2.5 mediates NO_3_^−^ transporter under low N conditions.

NRT2.5 is evolutionarily conserved across diverse organisms and is initially characterised as a NO_3_^−^ transporter responsive to nitrogen limitation (Kotur and Glass 2015). In wheat, *TaNRT2.5* is involved in NO_3_^−^ accumulation in the seeds in addition to responding to N starvation and NO_3_^−^ uptake, influencing seed viability and NO_3_^−^ distribution during reproductive stages (Li et al. 2020). In maize, *ZmNRT2.5* expression is induced under N starvation, and gene expression is detected in the roots, leaves, rachis, male ears and pericarp (Dechorgnat et al. 2019). In rice, *OsNRT2.3* is a homologue of *AtNRT2.5* and there are two transcripts, that is, *OsNRT2.3a* and *OsNRT2.3b* (Wang et al. 2021). OsNRT2.3a plays an important role in the transport of NO_3_^−^ from soil to plants, while OsNRT2.3b is capable of promoting N accumulation and transport within plants (Yan et al. 2011; Tang et al. 2012). In apple, long‐term low N treatment results in significant phenotypic and physiological advantages in *MdNRT2.5* overexpression lines, including an increased number of new leaves, more prominent root elongation, higher root and shoot dry weights, elevated total N content and root NO_3_^−^ content, as well as a higher ^15^NO_3_^−^ uptake rate (Hu et al. 2025). These results provide a key gene target for breeding economic crops with high N use efficiency, which is expected to promote molecular breeding research of related crops.

Soil salinization is one of the world's major environmental disasters, leading to reduced crop yields and huge economic losses. Effective use of large areas of saline soil resources is crucial for ensuring food security (Song and Wang 2015). In saline environments, N often serves as a key nutrient that limits plant growth. Therefore, enhancing N fertilisation emerges as an effective strategy to promote plant development under salt stress. For the majority of plants, high salinity adversely affects N uptake, transport, and subsequent assimilation (Kaiser et al. 2000). For example, 75 mmol L^−1^ NaCl treatment significantly downregulated the *PsNRT2.5* expression in *Populus simonii* (Zhang et al. 2014). Using non‐invasive microtomography and ^15^NO_3_^−^ tracking techniques, it is found that 100 mM NaCl significantly reduced NO_3_^−^ uptake and transport in salt‐sensitive *Populus popularis*, and that the expression of *NRT1.1*, *1.2*, *2.4* and *3.1* is significantly down‐regulated, but a completely opposite trend is observed in salt‐tolerant *P. euphratica* (Liu et al. 2024). Seagrass ( *Zostera marina* ) *NRT2.1* has a high degree of sequence and functional similarity to *NRT2.5*, and it plays a role in NO_3_^−^ uptake when there is prolonged N starvation or extremely low NO_3_^−^ concentrations (Rubio et al. 2019; Lezhneva et al. 2014). *ZosmaNRT2.1* is a NO_3_^−^ transporter with high‐affinity Na^+^ as the driver ion, which may be necessary for seagrass adaptation to the marine environment (Rubio et al. 2019). In the highly salt‐tolerant halophyte *Salicornia europaea* , proteomics analysis of the root plasma membrane revealed that NaCl promotes NO_3_^−^ uptake (Nie et al. 2015). These results suggest that the mechanisms of NO_3_^−^ uptake and use under salinity may be different in salt‐sensitive and salt‐tolerant plants.

Promoters are switches that precisely control gene expression and are crucial elements in the regulation of gene expression (Rombauts et al. 2003; Palavecino et al. 2020). *Cis*‐acting elements have the capacity to exert a significant influence on the precise sensitivity and specificity of transcriptional responses (Potenza et al. 2004; Misra and Ganesan 2021). For example, GATA‐type transcription factors are present in higher plants and have been implicated in the regulation of N use in these plants (Bi et al. 2005). The nitrate‐responsive element (NRE; 5′‐(A/T)7A(C/G)TCA‐3′) is isolated from the nitrate reductase genes *NIA1* and *NIA2* (Konishi and Yanagisawa 2010). Adversity‐associated promoters can influence the expression of target genes in response to external stresses, helping plants to maintain normal growth and development under adverse conditions. Adversity‐related *cis*‐acting elements, such as the salt stress response element GT‐1, have been shown to be salt‐inducible in the promoter of soybean *SCAM4*. A salt‐inducible promoter fragment containing this element is also identified in *Suaeda liaotungens*is (Park et al. 2004; Li et al. 2016). The DRE element is involved in the regulation of salt stress in the regulation of a variety of adversity‐responsive genes, with the DRE response element in the promoter of *rd29A* in *Arabidopsis* (Narusaka et al. 2003). In rice, the DREB in the promoter is involved in the regulation of drought and high salinity (Yamaguchi‐Shinozaki and Shinozaki 2006). ABRE is an abscisic acid response element, and the ABRE element of *Arabidopsis rd29A/B* has been shown to be involved in the regulation of salt stress (Narusaka et al. 2003). In addition, promoter *c*is‐acting elements such as NAC/NAM, Myb/SANT, WRKY, VOZ, bHLH and AP2/ERE, which are transcription factor‐bound, act together to regulate downstream stress‐resistant gene expression during the regulation of both abiotic stresses (e.g., drought, salt) and hormonal signalling pathways (e.g., ABA) (Chinnusamy et al. 2004).

*Suaeda salsa* L. is a salt‐tolerant halophytic herb with succulent leaves, which can be used in developing saline agriculture (Song and Wang 2015). *S. salsa* is widespread in saline‐alkaline soils and is considered an excellent resource for environmental remediation. Meanwhile, it has high value as a vegetable, a source of medicine and feed for developing saline agriculture (Li et al. 2023). *S. salsa* exhibits strong salt tolerance and is often used as a model organism for plant salt tolerance studies (Song and Wang 2015). Song, Chen, et al. (2009) found that *S. salsa* leaves accumulate high concentrations of NO_3_^−^ in natural habitats. Furthermore, laboratory studies showed that high salinity (600 mM NaCl) did not significantly affect leaf NO_3_^−^ content, regardless of NO_3_^−^–N levels (Song, Chen, et al. 2009). Under low NO_3_^−^ conditions, 200 mM NaCl significantly enhanced the influx of ^15^NO_3_^−^ and NO_3_^−^ into the roots, resulting in increased NO_3_^−^ levels in both roots and leaves (Liu, Cui, Jia, et al. 2021; Liu, Cui, Lu, et al. 2021). Therefore, *S. salsa* can well adapt to low NO_3_^−^ and high salt habitat, indicating that *S. salsa* may have an efficient NO_3_^−^ uptake capacity.

We hypothesize that the functionality of *NRT2.5* and its promoter in the halophyte *S. salsa* may be different from non‐halophytes such as *Arabidopsis*. In this study, *S. salsa SsNRT2.5* and its promoter were cloned, and their functional properties related to NO_3_^−^ uptake were subsequently analysed using transgenic *Arabidopsis* and rice plants. Additionally, the NO_3_^−^ transport characteristics were investigated in rice plants with transferred *SsNRT2.5* and its promoter to elucidate the physiological and molecular genetic mechanisms by which NaCl enhances the efficient NO_3_^−^ transport in halophyte. This study will advance the exploration of gene resources, providing a theoretical framework and genetic materials for breeding N efficiency and salt‐tolerant crops, ultimately facilitating the effective utilisation of large areas of saline land.

## 2 Results

### 2.1 Analysis of the Gene and Promoter Sequence of SsNRT2.5

**FIGURE 1:**
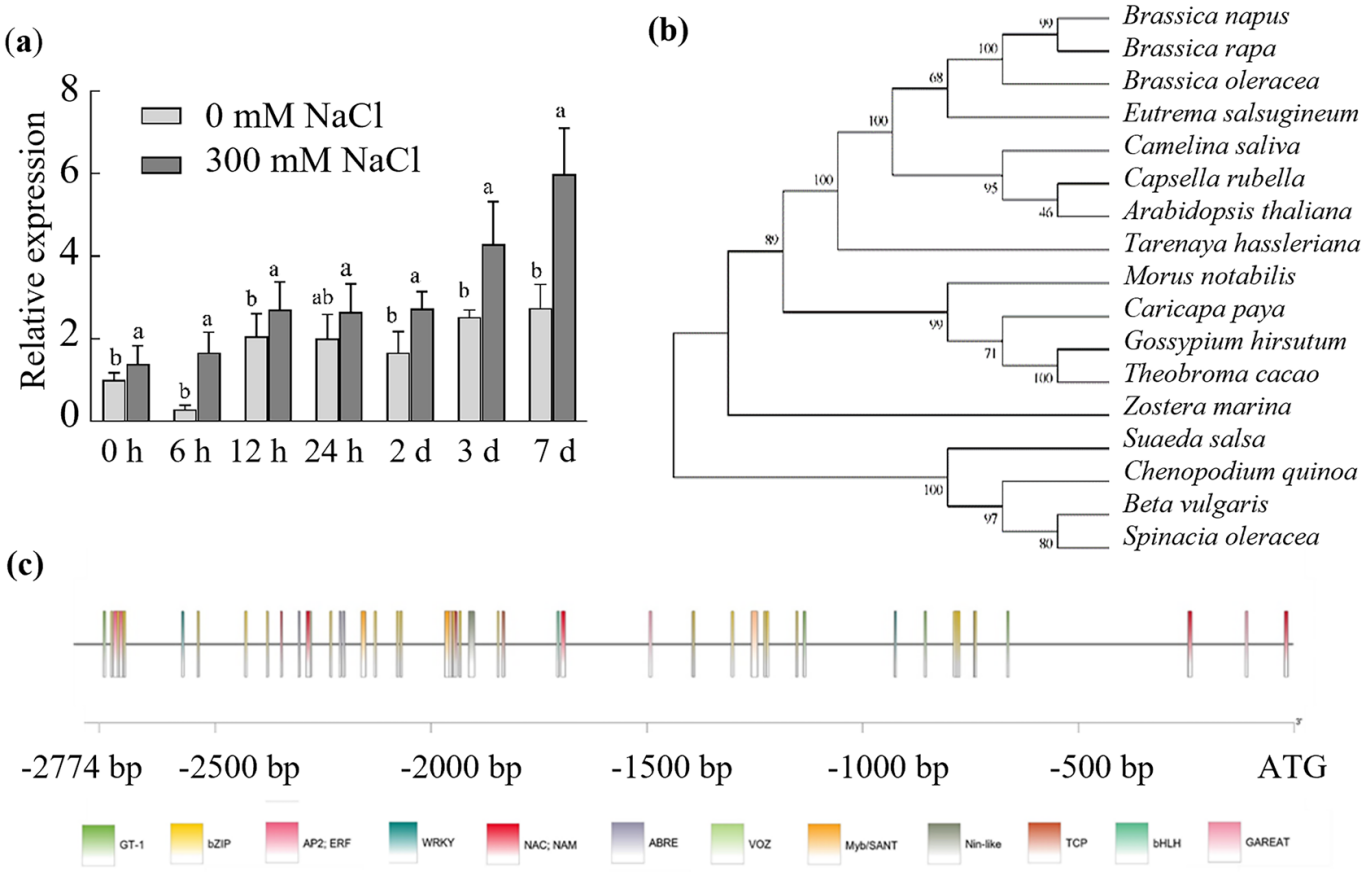
Sequence analysis and expression patterns of *SsNRT2.5* and its promoter. (a) Expression analysis of *SsNRT2.5* under salt stress in the roots; (b) Phylogenetic analysis of SsNRT2.5 with orthologs from other plant species; (c) Distribution of *cis*‐acting elements in the SsNRT2.5 promoter.

The putative cDNA sequence encoding the *SsNRT2.5* gene was isolated from the roots of *S. salsa* , with a full‐length sequence of 1653 bp (Table S1). The sequence exhibited a complete open reading frame (ORF*)* of 1524 bp. To investigate the evolutionary relationship of NRT2.5 proteins in different plant species, phylogenetic analysis revealed that SsNRT2.5 is most closely related to its orthologs from *Beta vulgaris* , *Chenopodium quinoa* , and *Spinacea oleracea* (Figure 1b).

To further elucidate the regulatory mechanism of *SsNRT2.5*, it is essential to obtain its promoter sequence. Using the genomic walking technique, a 2774 bp 5′ upstream sequence of the *SsNRT2.5* was successfully cloned. The *cis*‐acting elements of the *SsNRT2.5* promoter were analysed using PlantCARE, PLACE and PlantPAN databases. In addition to core elements (TATA box and CAAT box), the promoter harboured numerous putative *cis*‐acting elements, including nitrogen metabolism‐related motifs (e.g., nitrate‐inducible AGTCA, GATABOX, NPR, and nitrogen‐associated DE motif, TATABOX5; Table S2). Stress‐responsive, phytohormone‐regulatory, and transcription factor (TF) binding sites were also identified, such as salt‐responsive GT‐1, drought/high salt/low temperature‐responsive MYBR and DRE, MYC2‐mediated jasmonate (JA) response elements (TGACG and CGTCA motifs), auxin‐responsive TGA, ABA‐regulatory ABRE/AAGAA, gibberellin‐responsive P‐box, and salicylic acid‐responsive SARE (Table S2). Transcription factor binding elements linked to salt stress (e.g., NAC/NAM, Myb/SANT, WRKY, VOZ, bHLH, AP2/ERE families) may regulate *SsNRT2.5* expression under salt treatment, while nitrogen metabolism‐related TFs (TCP, bZIP, Nin‐like families) may mediate its expression under low nitrogen conditions (Figure 1c; Table S2).

The *AtNRT2*.*5* ORF (1506 bp) was isolated from *Arabidopsis* roots, together with its 2193 bp 5′ upstream sequence (Table S1). Promoter sequence comparison between *S. salsa* and *Arabidopsis* revealed shared *cis*‐elements related to salt stress, nitrogen metabolism, and other processes, but with significant quantitative differences. ProSsNRT2.5 contained more salt‐responsive GT‐1, nitrate‐inducible GATABOX, and ABA‐regulated ABRE/MYC elements than ProAtNRT2.5 (Table S2). Notably, nitrogen‐associated DE motif and multi‐stress‐responsive DRE were exclusive to ProSsNRT2.5.

### 2.2 Relative Expression Level of *SsNRT2.5* in the Roots and Seeds

The expression pattern of *SsNRT2.5* in *S. salsa* roots was significantly affected by salt stress (Figure 1a). Under 300 mM NaCl treatment, *SsNRT2.5* expression showed progressive upregulation over time and remained elevated for 7 days of salt treatment. In contrast, the expression stabilised at 12 h in the roots of *S. salsa* at 0 mM NaCl condition. Notably, the *SsNRT2.5* expression in the 300 mM NaCl‐treated group was consistently and significantly higher than those in the control at all time points examined (Figure 1a).

Additionally, regarding seeds of *S. salsa* in natural habitats, the relative expression of *SsNRT2.5* in black seeds was significantly higher than that in brown seeds (Figure S1a). Under 200 mM NaCl treatment, black seeds from salt‐treated mother plants exhibited significantly higher *SsNRT2.5* expression than brown seeds, which was correlated well with the observed NO_3_^−^ accumulation in seeds (Figure S1).

### 2.3 Subcellular Localization of SsNRT2.5 in *N. Benthamiana* and Expression Pattern of *SsNRT2.5* in *Arabidopsis*

**FIGURE 2:**
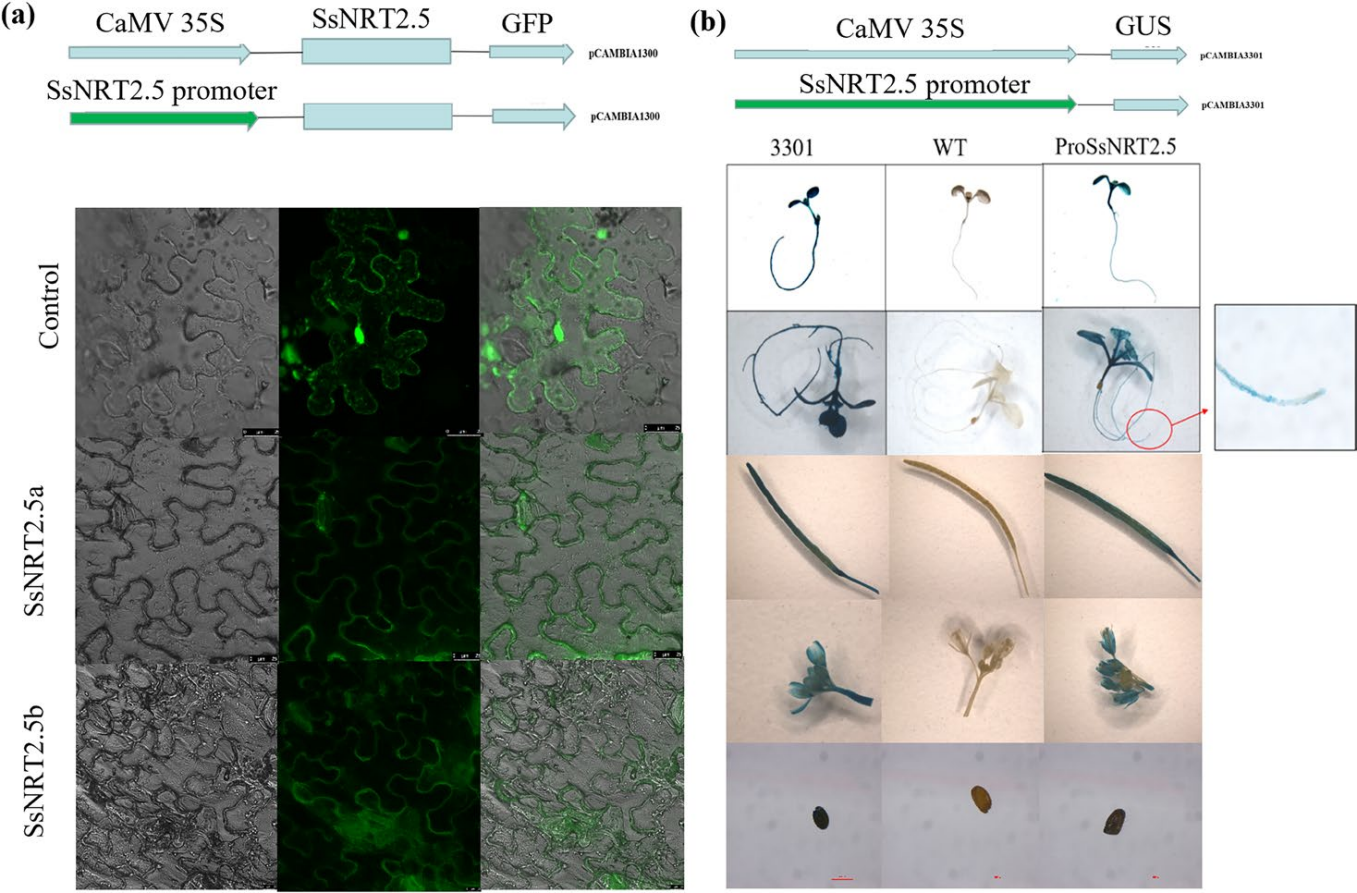
Subcellular localization and tissue‐specific expression of SsNRT2.5. (a) Subcellular localization of SsNRT2.5 in *Nicotiana benthamiana* leaf cells. The fusion constructs 35S::SsNRT2.5‐sGFP (SsNRT2.5a), pSsNRT2.5::SsNRT2.5‐sGFP (SsNRT2.5b), and 35S::sGFP (control) were agroinfiltrated into *N. benthamiana* leaves. Fluorescence signals were observed under a confocal microscope; (b) Tissue‐specific GUS activity driven by the SsNRT2.5 promoter in *Arabidopsis*. GUS staining was performed at different growth stages: Seedling (cotyledon stage, true leaf stage) and reproductive stage. 3301, empty vector control; WT, wild‐type *Arabidopsis*; ProSsNRT2.5, transgenic *Arabidopsis* harboring pSsNRT2.5::GUS. Representative images of GUS staining in the roots, cotyledons, true leaves, flowers, siliques, and seeds were shown.

To determine the subcellular localization of SsNRT2.5, the fusion constructs 35S::*SsNRT2.5‐*sGFP and pSsNRT2.5::*SsNRT2.5‐*sGFP, along with the control 35S::sGFP, were transiently transformed into leaf cells of *N. benthamiana* via agroinfiltration. The 35S::*SsNRT2.5* and pSsNRT2.5::*SsNRT2.5‐*sGFP were only detected in the plasma membrane (Figure 2a), indicating that SsNRT2.5 is a plasma membrane‐localised protein.

To analyze the *SsNRT2.5* expression pattern in different tissues, a 2774 bp upstream region of the *SsNRT2.5* coding region was fused to the GUS reporter gene and introduced into *Arabidopsis*. GUS staining at different growth stages of transgenic *Arabidopsis* showed that *SsNRT2.5* was mainly found in the roots, leaves, flowers, fruit pods and seeds (Figure 2b).

### 2.4 Fresh Weight, Root Length and NO_3_ ^−^ Content in Different *Arabidopsis* Lines Under Low Nitrogen Conditions

**FIGURE 3:**
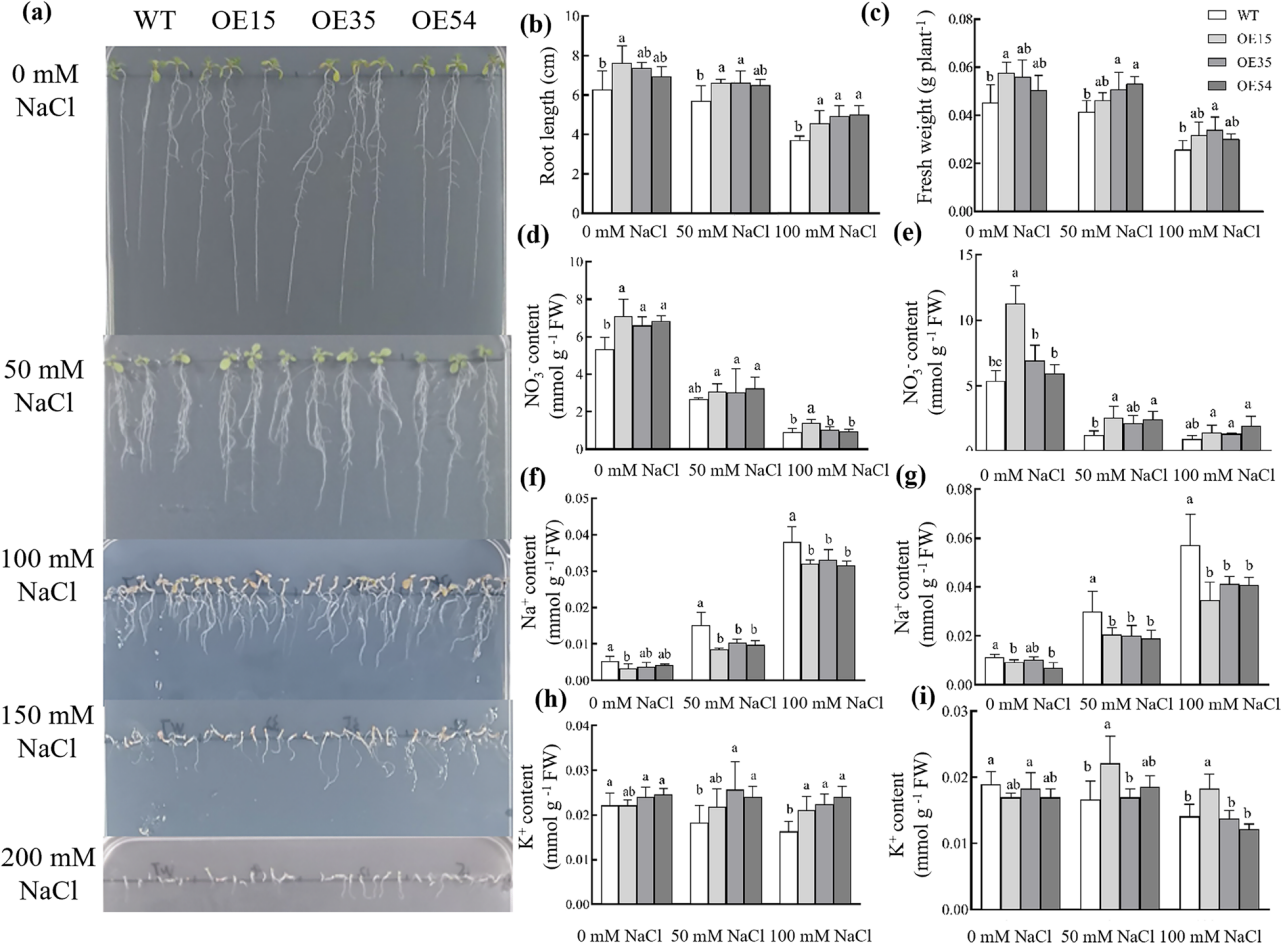
Phenotypic analysis of *SsNRT2.5*‐overexpressing *Arabidopsis* under salt stress. (a) Phenotypic comparison of WT and overexpression lines (OE15, OE35 and OE54) under different concentrations of NaCl; (b) Root length quantification; (c) Fresh weight measurement; Nitrate (NO_3_^−^) content in the roots (d) and leaves (e); Na^+^ content in the roots (f) and leaves (g); K^+^ content in the roots (h) and leaves (i).

To investigate the physiological function of *NRT2.5*, the 35S::*SsNRT2*.*5‐*sGFP overexpression vector was constructed, and *Arabidopsis* overexpression lines (OE15, OE35, OE54) were obtained. Meanwhile, *atnrt2.5* mutant (M2, M22) lines were generated in *Arabidopsis* using CRISPR/Cas9‐mediated gene editing (Figure S2). When treated with 0, 50, and 100 mM NaCl, the root length and fresh weight in OE15, OE35, and OE54 were significantly higher than those of WT (Figure 3a–c). Under 150 and 200 mM NaCl treatments, the plants were subjected to severe salt stress, and only the root length of OE35 was still significantly better than that of other lines, and the difference in fresh weight was no longer significant (Figure 3a,b).

Under 0 mM NaCl treatment, the NO_3_^−^ content in the roots and leaves of the overexpression lines were significantly higher than that of WT; after 50 mM NaCl treatment, the NO_3_^−^ content of all lines decreased, but the decrease in WT was more obvious, and OE15, OE35, and OE54 still maintained a relatively high level (Figure 3d,e); under 100 mM NaCl treatment, only the NO_3_^−^ content in the roots of OE15 was significantly higher than that of WT (Figure 3d,e). Under 0 mM NaCl treatment, the overexpression lines exhibited slightly lower Na^+^ content but a significantly higher K^+^ content compared to the WT (Figure 3f–i); after salt treatment, the Na^+^ content of both WT and the overexpression lines increased significantly, but the Na^+^ accumulation in the overexpression lines was significantly lower than that in WT (Figure 3f,g); the K^+^ content in the roots of the overexpression lines was always higher than that of WT (Figure 3h,i).

**FIGURE 4:**
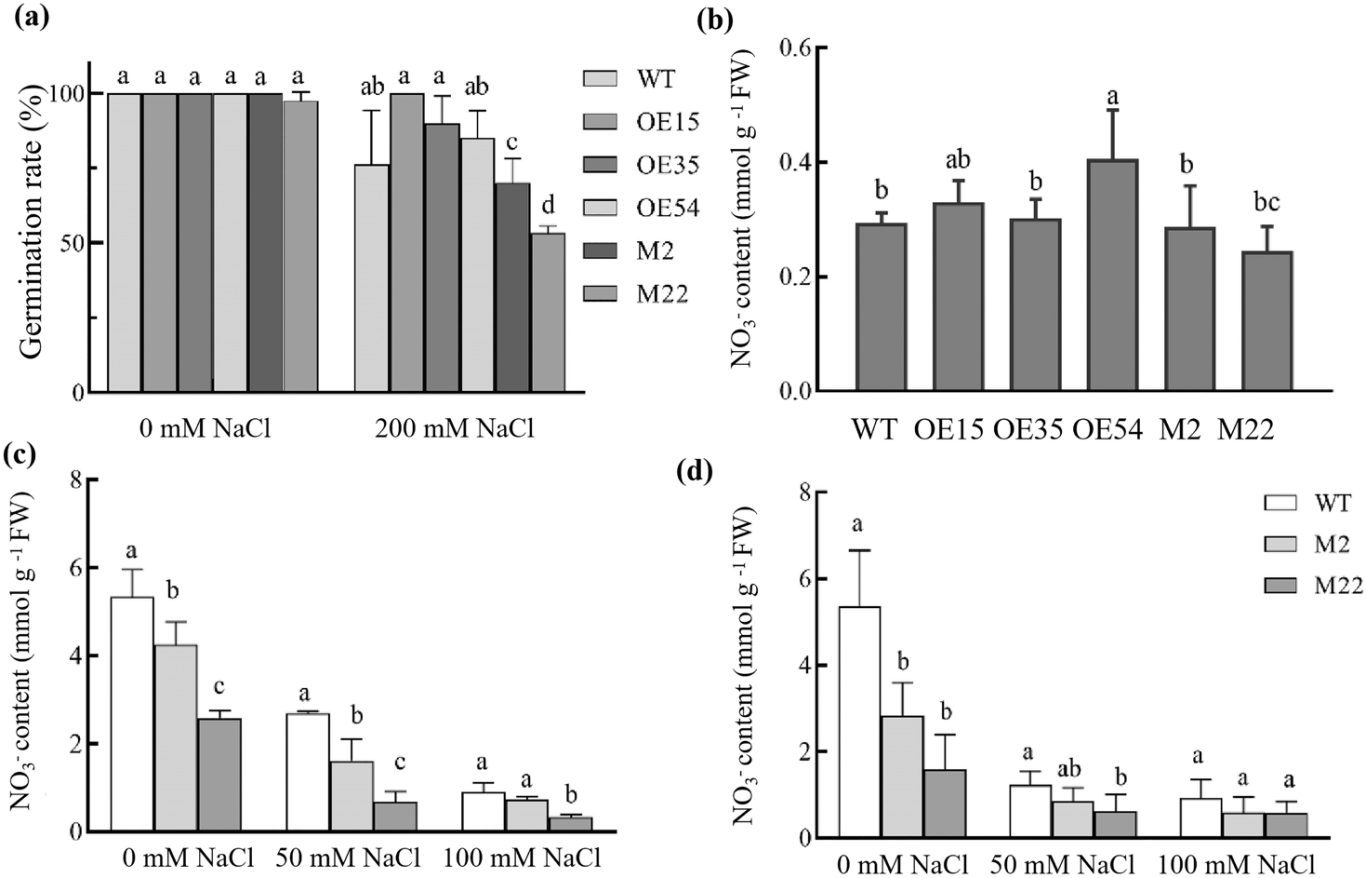
NO_3_^−^ content in *Arabidopsis* seeds and germination percentage under different NaCl concentrations. (a) Germination percentage under 0 and 200 mM NaCl; (b) NO_3_^−^ content in seeds of WT, overexpression lines (OE15, OE35 and OE54) and knockout mutants (M2 and M22); (c, d) NO_3_^−^ content in the roots and leaves under 0, 50 and 100 mM NaCl treatment.

Under salinity combined with either 0.5 or 0.1 mM NO_3_^−^, the growth of M2 and M22 showed no significant difference from that of WT (Figure S3). However, under 0 mM NO_3_^−^ treatment, the root length and fresh weight of M22 were significantly lower than those of WT (Figure S3). Under low NO_3_^−^ conditions (0.5 mM), at 0 mM NaCl, the germination percentage of the overexpression lines was comparable to that of *atnrt2.5* lines, with a success rate of nearly 100% (Figure 4a). At 200 mM NaCl treatment, except for OE15, the germination percentages of all lines decreased. The germination percentage of the WT was approximately 70%, and that of M2 and M22 was approximately 60% and 50%, respectively (Figure 4a). Compared with WT, the NO_3_^−^ content in the seeds of overexpression lines OE15 and OE54 was significantly increased. In contrast to WT, the NO_3_^−^ content in the seeds of M22 was decreased (Figure 4b). This phenomenon is probably associated with the relatively higher accumulation of NO_3_^−^ in the seeds of *Arabidopsis* overexpression lines. This means elevated concentrations of NO_3_^−^ can enhance the salt tolerance of seeds and improve the germination percentage. The NO_3_^−^ content in the roots and leaves of the mutant lines was significantly lower than that of the WT, and the reduction in NO_3_^−^ content was more pronounced in the mutants under high salinity (Figure 4c,d).

### 2.5 Responses of *SsNRT2.5* to Salt and Low Nitrate Driven by Self‐Promoter

**FIGURE 5:**
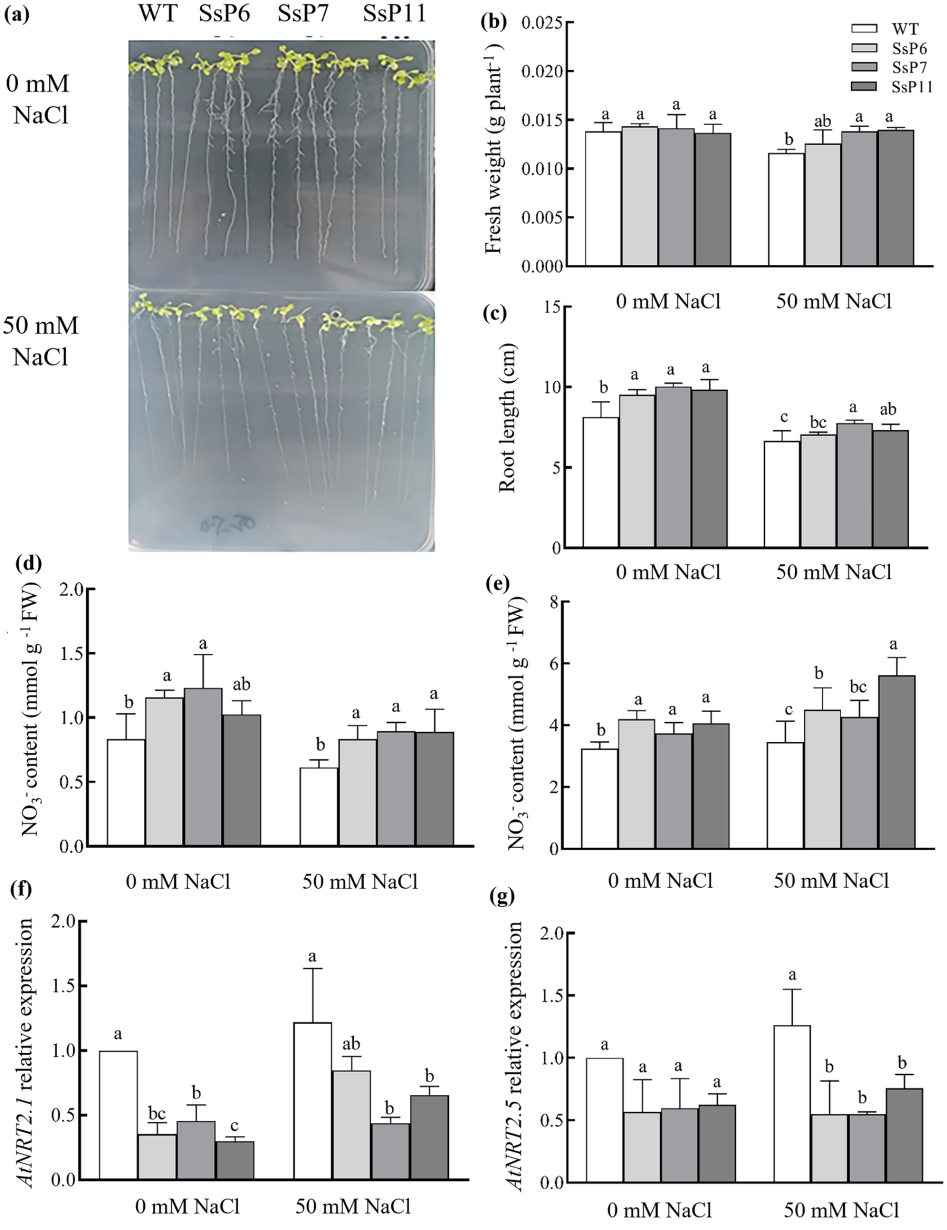
Salt‐induced changes in growth and NO_3_^−^ accumulation of *Arabidopsis* transgenic lines harbouring pSsNRT2.5::*SsNRT2.5*‐sGFP under 0.5 mM NO_3_^−^ condition. (a) Phenotypes of WT and transgenic lines (SsP6, SsP7 and SsP11) grown on solid medium with 0.5 mM NO_3_^−^ under 0 and 50 mM NaCl treatments; Fresh weight (b), root length (c), NO_3_^−^ contents in the roots (d) and leaves (e), *AtNRT2.1* (f) and *AtNRT2.5* (g) relative expression of WT and transgenic lines under 0 and 50 mM NaCl treatments.

To elucidate the regulatory role of the *SsNRT2.5* promoter in mediating NO_3_^−^ uptake under salt stress, a pSsNRT2.5::*SsNRT2*.*5‐*sGFP vector was constructed, and *Arabidopsis* transgenic lines were obtained. On low‐nitrate (0.5 mM) solid medium without salt stress (0 mM NaCl), the transgenic lines (SsP6, SsP7, and SsP11) showed no significant difference in fresh weight compared to the WT (Figure 5a). However, under 50 mM NaCl treatment, the SsP6, SsP7, and SsP11 transgenic lines exhibited increased fresh weight and root length (Figure 5b,c). Moreover, the NO_3_^−^ content in the roots and leaves of the SsP6, SsP7, and SsP11 was significantly higher than that of the WT (Figure 5d,e). Furthermore, compared with the 0 mM NaCl treatment, the NO_3_^−^ content in the leaves was also increased in the SsP6, SsP7, and SsP11 under 50 mM NaCl treatment (Figure 5e). This indicates that the transgenic lines possess an enhanced capacity for NO_3_^−^ uptake, which can be further promoted by moderate salt stress (50 mM NaCl), leading to elevated NO_3_^−^ accumulation.

In 1/2 MS medium, under 100 and 150 mM NaCl treatments, the root length and fresh weight of the SsP6, SsP7, and SsP11 lines were observed to be greater than those of the WT (Figure S4). In nutrient soil, at the vegetative growth stage, 100 and 150 mM NaCl treatments significantly increased the biomass and leaf NO_3_^−^ content of the SsP6, SsP7, and SsP11 lines (Figure S5a–c,g), decreased the Na^+^ content, with no significant change in K^+^ content (Figure S6a,b), and decreased MDA content (Figure S6c). The expression of *AtHKT1* was increased (Figure S6d). At the reproductive growth stage, under 100 mM NaCl treatment, the biomass and seed NO_3_^−^ content of the SsP6, SsP7, and SsP11 lines exhibited a notable increase in comparison to the WT (Figure S5d–f, h). This indicated that the transgenic lines exhibited enhanced tolerance to salt stress, accompanied by an elevated level of NO_3_^−^ accumulation in the leaves and seeds.

### 2.6 Comparative Analysis of the *NRT2.5* Gene in Response to Salt Stress Between *S. salsa* and *Arabidopsis*

Under both 0 and 100 mM NaCl conditions, *Arabidopsis* lines overexpressing either *SsNRT2.5* or *AtNRT2.5* exhibited significantly longer roots than the WT at the seed germination stage when grown on low nitrate (0.5 mM NO_3_^−^) medium (Figure S7a). Among the transgenic lines, overexpression of *SsNRT2.5* led to the longest roots, and a more pronounced effect was observed under 150 mM NaCl treatment (Figure S7a,c). At the seedling stage, under low‐nitrogen hydroponic conditions, both 0 and 50 mM NaCl treatments significantly increased the NO_3_^−^ content of all overexpression lines; under 100 mM NaCl treatment, the leaf NO_3_^−^ content in the lines overexpressing *SsNRT2.5* significantly increased compared to lines overexpressing *AtNRT2.5* (Figure S7g,h). Under 50 and 100 mM NaCl treatments, the biomass was significantly higher and the leaf Na^+^ content was significantly lower in each overexpression line compared to the WT (Figure S7b–f). The SsOE15 and SsOE35 showed the most vigorous growth and the lowest leaf Na^+^ content (Figure S7f). The aforementioned results indicated that the SsOE15 and SsOE35 lines exhibited a superior NO_3_^−^ uptake capacity compared to the AtOE4 and AtOE6 lines (Figure S7g,h).

### 2.7 Comparative Analysis of the *NRT2.5* Driven by Self‐Promoter in Response to Salt Stress Between *S. salsa* and *Arabidopsis*

**FIGURE 6:**
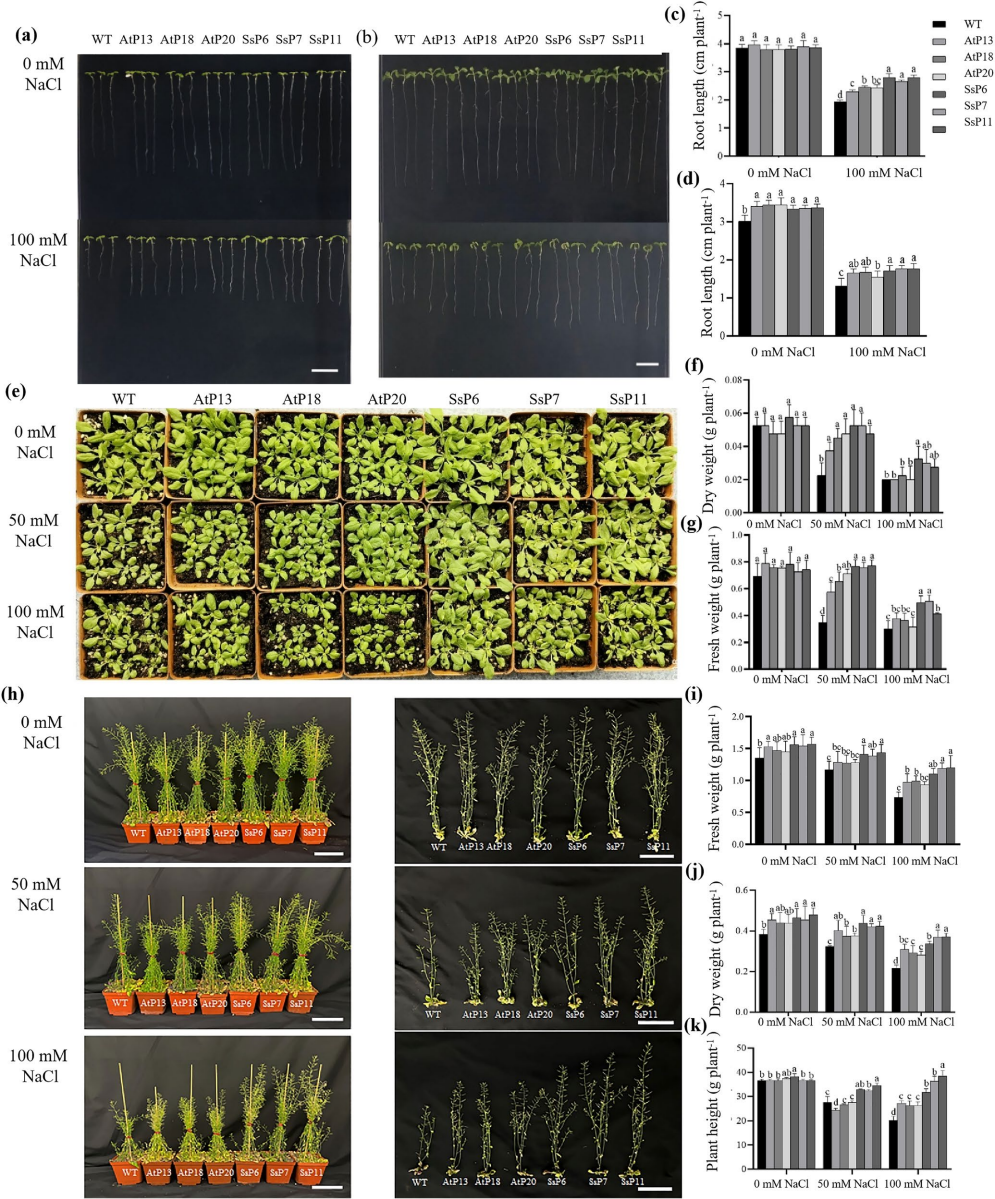
Comparison of salt tolerance between *Arabidopsis* transgenic lines harbouring *SsNRT2.5* with its promoter and *AtNRT2.5* with its promoter under salt stress. (a–d) Phenotype and root length at the seed germination stage under 0.5 mM NO_3_^−^ (a, c) and ½ MS (b, d) with 0 and 100 mM NaCl; (e–g) Phenotype and changes in fresh and dry weight under 0.5 mM NO_3_^−^ combined with 0, 50, and 100 mM NaCl at the seedling stage; (h–k) Phenotype and changes in fresh and dry weight, plant height under 0.5 mM NO_3_^−^ combined with 0, 50, and 100 mM NaCl at the reproductive growth stage.

**FIGURE 7:**
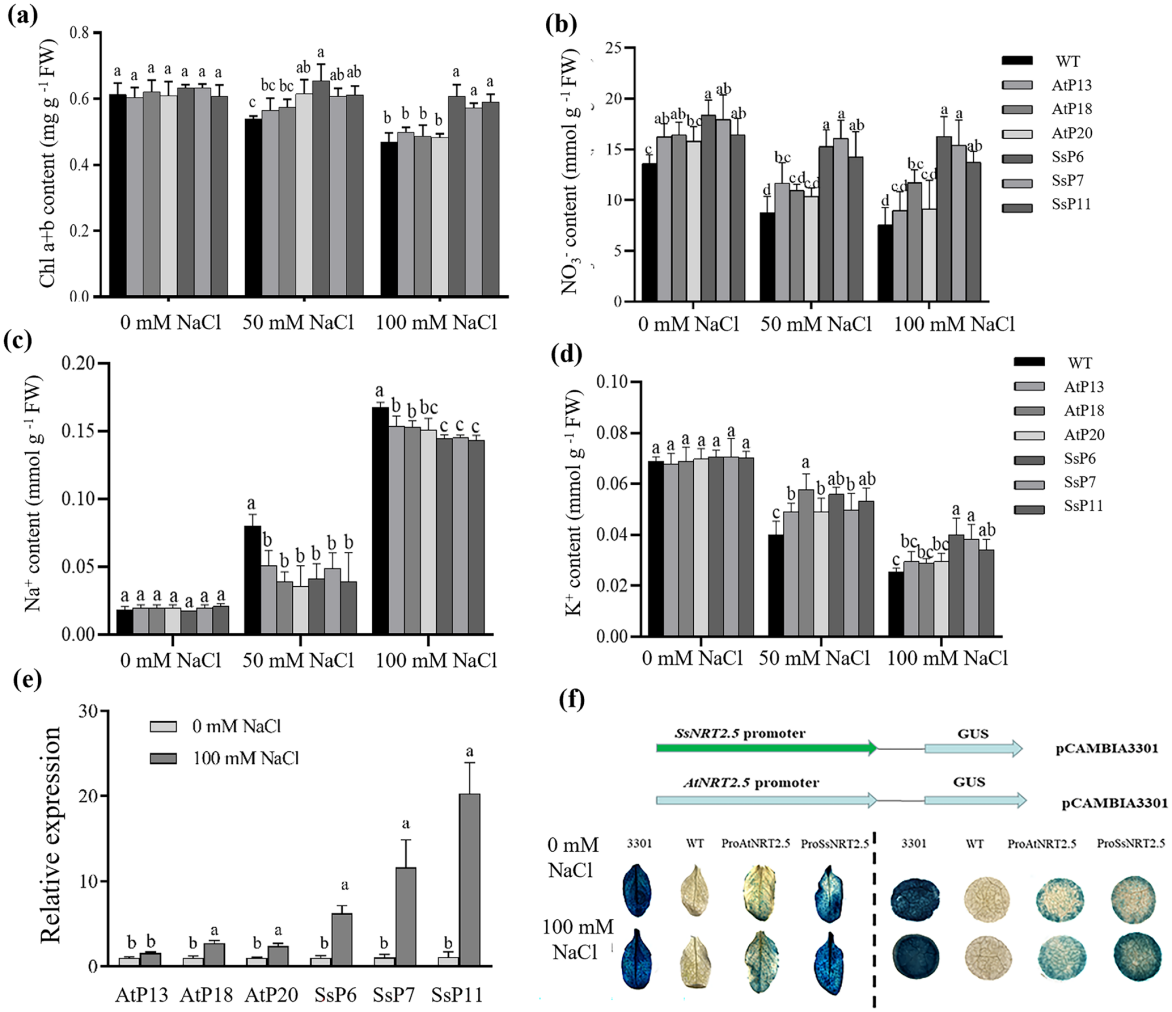
Physiological responses and promoter function analysis of *Arabidopsis* transgenic lines with *NRT2.5* and its promoter under salt stress. (a–d) Changes in chlorophyll, NO_3_^−^, Na^+^ and K^+^ content in *Arabidopsi*s lines transduced with pAtNRT2.5::*AtNRT2.5‐*sGFP, pSsNRT2.5::*SsNRT2.5*‐sGFP and WT under 0, 50 and 100 mM NaCl treatments; (e) Relative expression levels of *NRT2.5* in each line under 100 mM NaCl treatment with low‐nitrogen conditions; (f) GUS staining results in the leaves of transgenic *Arabidopsis* and transiently transformed *N. benthamiana*, comparing the expression differences of GUS reporter gene driven by ProSsNRT2.5 and ProAtNRT2.5.

A functional comparison was conducted on pAtNRT2.5::*AtNRT2.5‐*sGFP and pSsNRT2.5::*SsNRT2.5‐*sGFP transgenic *Arabidopsis* lines under low nitrogen condition. In 1/2 MS medium, no significant difference in root length was observed among the lines under 0 mM NaCl (Figure 6b). Under 100 mM NaCl treatment, all transgenic lines showed an increase in root length, with the most significant increase observed in the SsP6, SsP7, and SsP11 lines (Figure 6a,b). At the seedling growth stage, compared with WT under salinity, the plant height, fresh and dry weight of seedlings, leaf NO_3_^−^, K^+^, and chlorophyll contents in transgenic lines were significantly increased, while the leaf Na^+^ content and relative electrical conductivity were decreased (Figures 6 and 7). At 100 mM NaCl, the changes in the above parameters in SsP6, SsP7, and SsP11 lines were more extensive than those in the AtP13, AtP18, and AtP20 lines (Figures 6 and 7). Under 100 mM NaCl treatment, the expression of *NRT2.5* was significantly increased in the transgenic lines compared to the WT, particularly in SsP6, SsP7 and SsP11 lines, in which the increase was most pronounced (Figure 7e). The aforementioned results demonstrated that *Arabidopsis* lines transfected with the *SsNRT2.5* gene and its promoter exhibited a markedly enhanced salt tolerance compared to those transfected with the *AtNRT2.5* gene and its promoter.

At the vegetative growth stage, GUS staining was performed on the leaves of the pAtNRT2.5::*GUS* and pSsNRT2.5::*GUS* transgenic *Arabidopsis* lines. It was observed that both transgenic lines showed deeper staining under 100 mM NaCl treatment (Figure 7f). Furthermore, the staining degree of the ProSsNRT2.5‐ transduced line was consistently higher than that of the ProAtNRT2.5 transduced line. Similar observations were found in transiently transformed tobacco plants (Figure 7f).

### 2.8 Effect of 5′ Deletions on the Activity of *SsNRT2.5* Promoter

**FIGURE 8:**
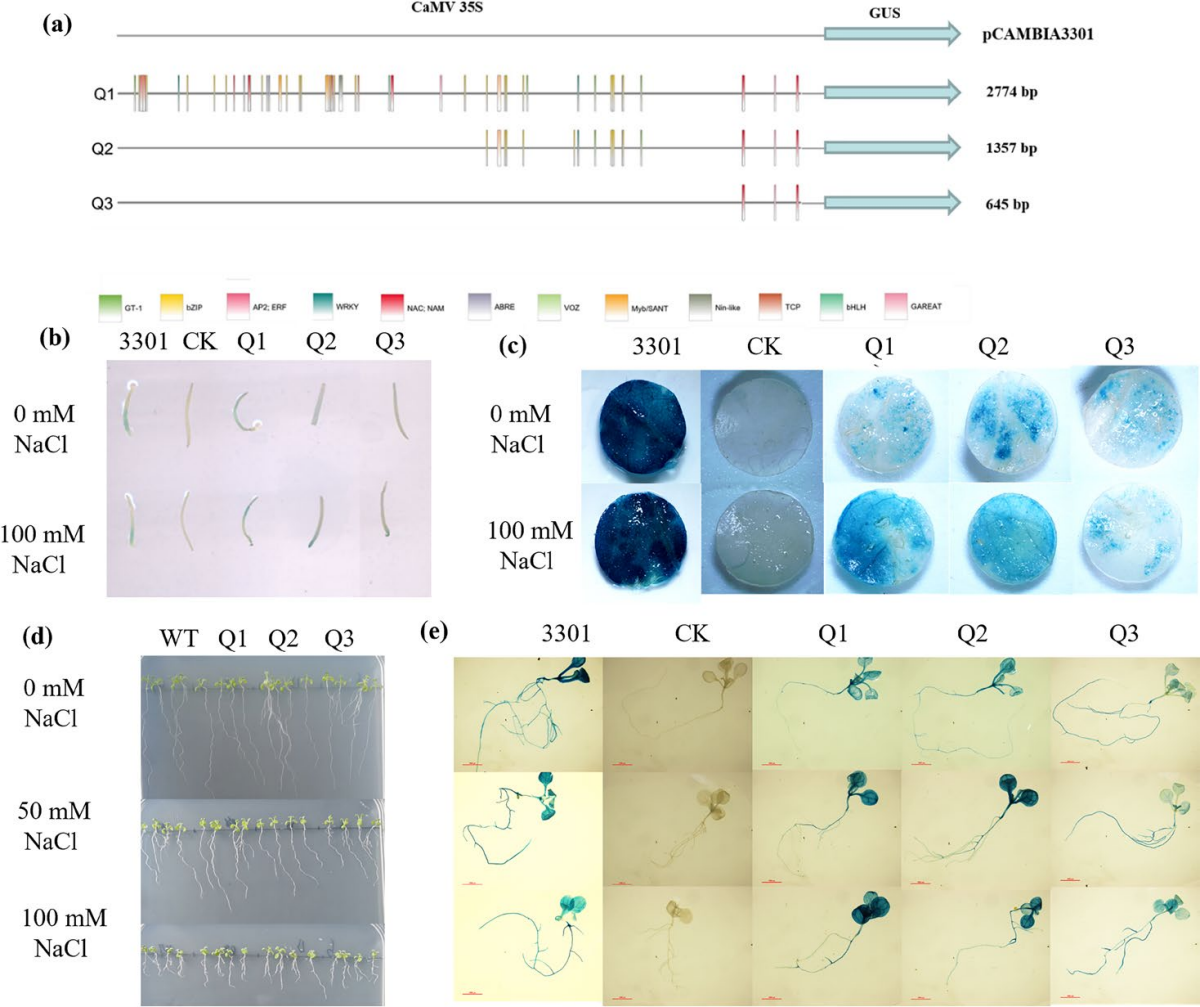
Functional characterisation of 5′‐deleted *SsNRT2.5* promoter fragments. (a) Schematic of deletion constructs Q1–Q3 in pCAMBIA3301 vector (CaMV 35S terminator); (b, c) GUS staining in transiently transformed *S. salsa* and *N. benthamiana* showing NaCl‐induced activation of Q1/Q2 segments; (d, e) GUS activity in transgenic *Arabidopsis* under salt treatment (WT: Wild‐type; CK: Empty vector control). Scale bars: 1 cm.

The 5′ deletion approach was employed to dissect the promoter function (Figure 8a). GUS activity was monitored via transient transformation in *S. salsa* and *N. benthamiana*. In *S. salsa* , GUS staining was enhanced in the Q1 and Q2 segments but attenuated in the Q3 segment under 100 mM NaCl treatment (Figure 8b). A comparable trend was observed in *N. benthamiana*, indicating that the promoter regions corresponding to Q1 and Q2 could be induced by NaCl (Figure 8c). In transgenic *Arabidopsis*, GUS staining results were consistent with those of transient transformation, i.e., salt stress enhanced GUS staining intensity in Q1 and Q2, suggesting that salt stress induced *GUS* gene expression (Figure 8d,e). These results demonstrated that the *SsNRT2.5* promoter is a salt‐inducible promoter; due to the presence of salt‐related *cis*‐acting elements in the Q1 and Q2 segments, the promoter could drive increased gene expression under salt stress.

### 2.9 Responses of *SsNRT2.5* to Salt Stress and Low Nitrate Driven by Self‐Promoter in Rice

**FIGURE 9:**
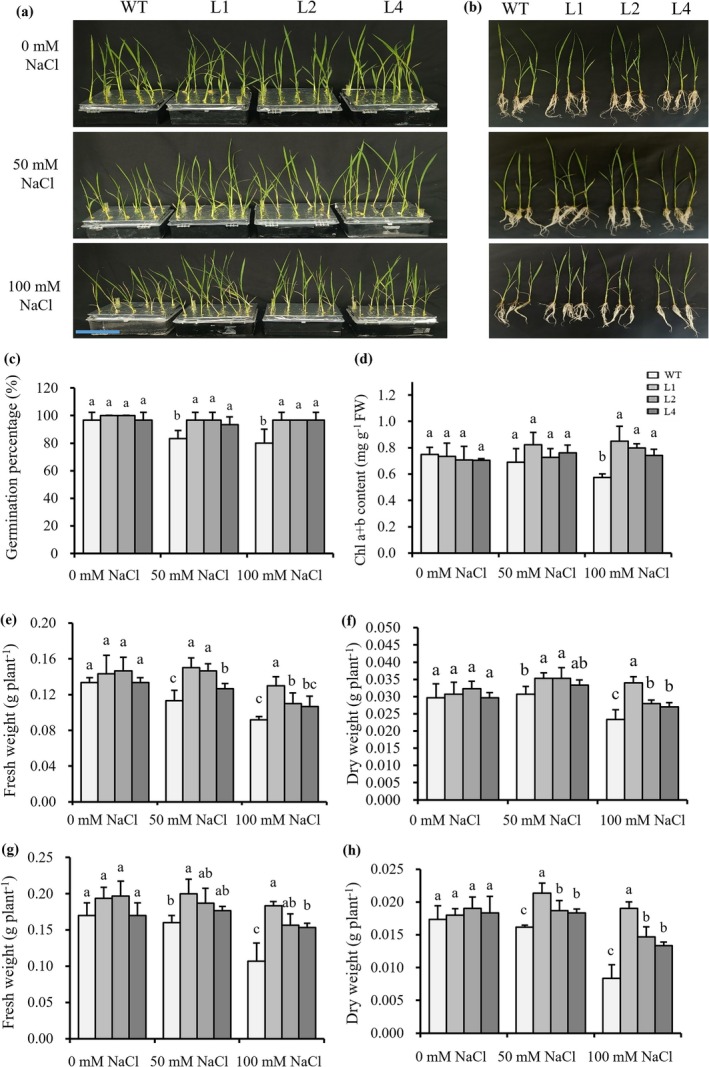
Effects of salinity on the growth and physiological characteristics of WT and pSsNRT2.5::*SsNRT2.5* transgenic rice lines. (a, b) Phenotypic observation at 0, 50, and 100 mM NaCl; (c) Germination percentage statistics; (d) Chlorophyll a + b content; Shoots (e,f) and roots (g,h) fresh and dry weight analysis.

**FIGURE 10:**
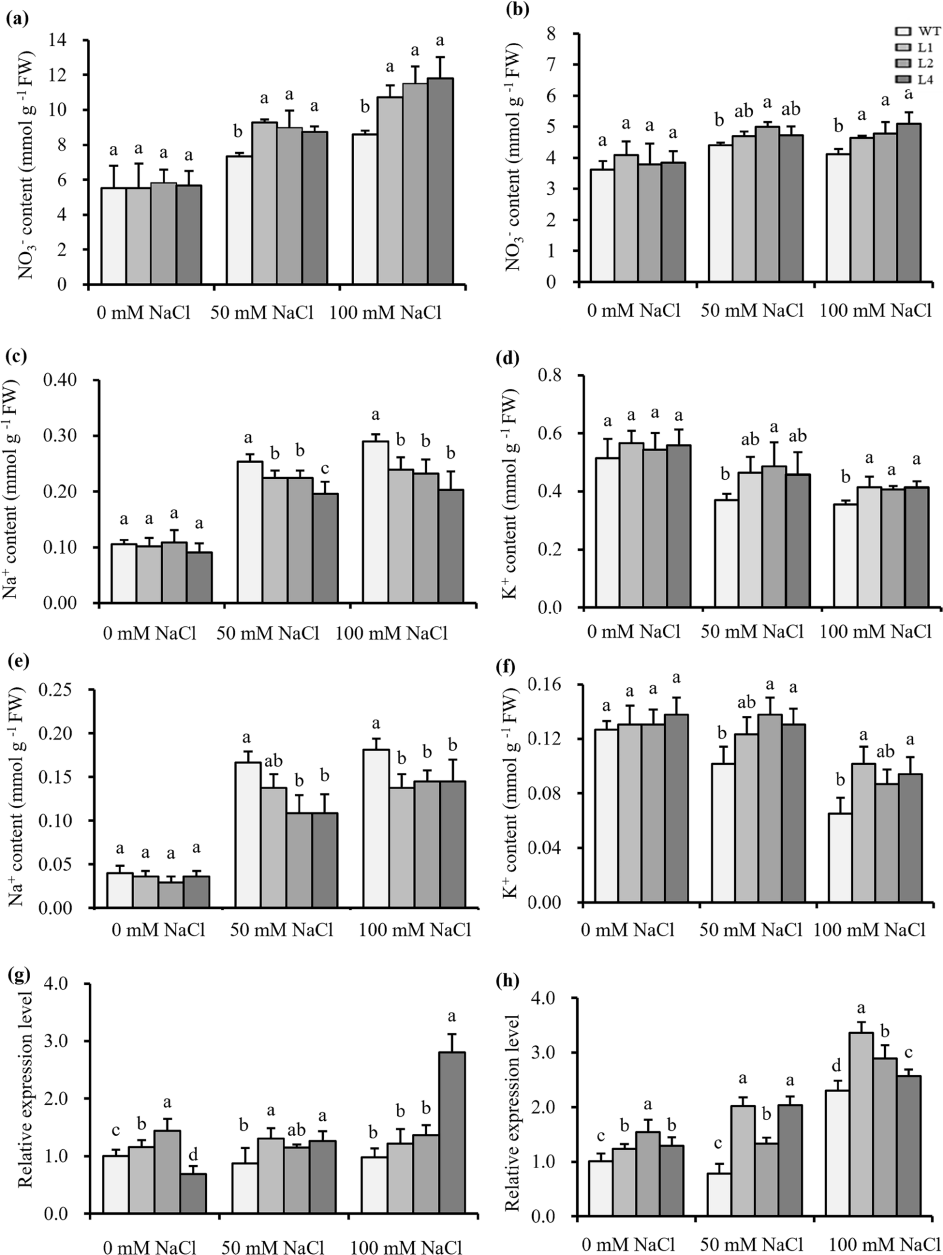
Ion homeostasis and gene expression responses of WT and pSsNRT2.5::*SsNRT2.5* transgenic rice lines under salt stress. (a, b) NO_3_^−^ content in the leaves and roots at 0, 50, and 100 mM NaCl. (c, d) Na^+^ and K^+^ content in the roots; (e, f) Na^+^ and K^+^ content in the leaves; (g, h) Relative expression of *OsHKT1* (g) and *OsSOS1* (h).

The pSsNRT2.5::*SsNRT2.5*‐GFP expression vector was constructed and introduced into *Oryza sativa* . No distinct differences in phenotype and parameters were observed between the transgenic rice lines (L1, L2, L4) and WT at 0 mM NaCl (Figures S8a,b and 9). However, at 50 and 100 mM NaCl, the germination percentage and fresh weight of the transgenic lines were significantly higher than those of WT (Figure 9). Under hydroponic conditions, 50 and 100 mM NaCl treatments led to remarkable phenotypic distinctions between the transgenic rice lines (L1, L2, L4) and the WT (Figure 9a,b), and 100 mM NaCl treatments led to a significant increase in chlorophyll a + b content in the transgenic rice lines (Figure 9d). Additionally, significant differences were detected in dry weight, fresh weight and leaf NO_3_^−^ content between WT and the transgenic rice lines (Figures 9e–h and 10a,b). Leaf Na^+^ content decreased significantly, while K^+^ content increased (Figure 10c,d), meanwhile, the expression levels of *OsHKT1* and *OsSOS1* in the roots of transgenic lines were all up‐regulated compared with WT (Figure 10g,h), indicating that the salt tolerance of rice was enhanced by the transfection of *SsNRT2.5*. Under 100 mM NaCl treatment, the seed yield per plant and seed NO_3_^−^ content were significantly higher in transgenic lines compared to WT (Figure S8d). The expression level of *SsNRT2.5* was higher in the seeds of the transgenic lines than in the WT (Figure S8e), suggesting its functional importance during seed development. Importantly, *SsNRT2.5* not only facilitates root NO_3_^−^ uptake but also mediates NO_3_^−^ translocation and storage in seeds. This dual functionality provides novel insights into plant nitrogen allocation mechanisms and offers valuable genetic resources for developing salt‐tolerant crops with improved nitrogen use efficiency.

## 3 Discussion

Salinity is one of the major abiotic stresses that adversely affects plant growth and development by inducing ion toxicity, osmotic stress, and nutrient imbalance (Song and Wang 2015). Nitrate, the primary nitrogen source for plants in natural environments, plays a critical role in salt tolerance. Previous studies have demonstrated that *S. salsa* exhibits efficient NO_3_^−^ uptake under saline conditions, suggesting the evolution of distinct NO_3_^−^ acquisition mechanisms in halophytes compared to NO_3_^−^ uptake in non‐halophytes (Liu, Cui, Jia, et al. 2021; Liu, Cui, Lu, et al. 2021). However, the molecular and genetic mechanisms regarding high‐efficiency N uptake in halophytes remain unclear. In the present study, the *SsNRT2.5* gene and its promoter were cloned from the halophyte *S. salsa* and performed a comparative analysis with *Arabidopsis AtNRT2.5* and its promoter, aiming to elucidate how *SsNRT2.5* and its promoter facilitate efficient NO_3_^−^ uptake in *S. salsa* under salinity and low NO_3_^−^ availability.

Previous evidence indicates that *SsNRT2.5* plays a pivotal role during N starvation, with its expression in old leaves significantly higher than that in young leaves under N‐deprived conditions (Ma et al. 2020). This study further reveals that salinity continuously upregulates *SsNRT2.5* expression under low NO_3_^−^–N (Figure 1a), contrasting with the expression pattern of *SsNRT2.1* (Liu, Cui, Jia, et al. 2021; Liu, Cui, Lu, et al. 2021). In *Arabidopsis*, *AtNRT2.5* functions as the primary high‐affinity nitrate transporter responding to extremely low NO_3_^−^ levels, acting in concert with *NRT2.1*, *NRT2.2*, and *NRT2.4* to coordinate the NO_3_^−^ uptake (Lezhneva et al. 2014). In the *SsNRT2.5* overexpression lines, *AtNRT2.5* and *AtNRT2.1* expression were downregulated (Figure 5f,g) likely due to negative regulation by high *SsNRT2.5* expression, consistent with Kotur and Glass (2015). Additionally, supplementing 0.5 mM NO_3_^−^ to nitrogen‐free medium increased fresh weight and root length in *SsNRT2.5* overexpression lines, accompanied by variable NO_3_^−^ accumulation in the roots and leaves under 0, 50 and 100 mM NaCl (Figure 3), indicating *SsNRT2.5* promotes NO_3_^−^ absorption under saline conditions, that is, it is involved in NO_3_^−^ uptake and transport in high‐salinity and low NO_3_^−^ environments.

Subcellular localization studies showed that SsNRT2.5 localized on the plasma membrane (Figure 2a), similar to AtNRT2.5 (Lezhneva et al. 2014). GUS staining is a tool to predict gene expression domains (Lezhneva et al. 2014). Expression of the pSsNRT2.5::*GUS* construct was observed in both roots and leaves (Figure 2b), consistent with *AtNRT2.5* expression in *Arabidopsis* (Lezhneva et al. 2014). During the reproductive stage, *SsNRT2.5* was expressed in the flowers, pods, and seeds, with overexpression lines accumulating higher seed NO_3_^−^ (Figures 2b and 4b). This study demonstrates that SsNRT2.5 regulates NO_3_^−^ accumulation in seeds, as evidenced by increased NO_3_^−^ in seeds of *Arabidopsis* overexpression lines (OE15, OE54) and reduced levels in the M22 mutant. In wheat, the TaNAC2‐NRT2.5 module enhances seed NO_3_^−^ content and germination by transcriptional activation, suggesting its potential for improving yield and N use efficiency (Li et al. 2020). Additionally, higher *SsNRT2.5* expression in black seeds of *S. salsa* correlates with elevated NO_3_^−^, supporting a conserved role in nitrate‐mediated seed vigour, which may contribute to salinity adaptation (Song et al. 2016).

Promoters are pivotal for regulating gene expression, serving as key components in gene activity control (Rombauts et al. 2003). Promoter activity indirectly reflects the expression of its target gene (Cazier and Blazeck 2021). While *cis*‐acting elements influence the sensitivity and specificity of transcriptional responses (Dansako et al. 2003). The *SsNRT2.5* promoter contains multiple salt‐stress‐related *cis*‐elements (e.g., GT‐1, DRE, DREB, ABRE, G‐box, W‐box, MYB recognition sites) alongside nitrogen‐responsive elements (Table S2), which indicates that it is salt‐inducible. We hypothesize that salt treatment may activate *SsNRT2.5* expression, maintaining high transcription under high salinity and low nitrogen, since *S. salsa* can accumulate large amount of NO_3_^−^ in the leaves (Song, Chen, et al. 2009; Song, Shi, et al. 2009). The full‐length *SsNRT2.5* promoter exhibits high activity, but gradually decreases with 5′ deletions (Figure 8), which is similar to the *CgbHLH001* promoter in *Chenopodium glaucum* , that is, the promoter activity declines with sequential 5′ deletions (Zhou et al. 2023). This demonstrates that *cis*‐elements within the *SsNRT2.5* promoter significantly influence its activity. Compared to *AtNRT2.5*, the *SsNRT2.5* promoter harbours a greater number of salt‐response and stress‐responsive transcription factor‐binding elements (Table S2), suggesting a more pronounced role in regulating NO_3_^−^ uptake under salt stress.

To assess the regulatory role of the *SsNRT2.5* promoter under salt stress, pSsNRT2.5::*SsNRT2.5*‐sGFP was transformed into *Arabidopsis*. Under 0.5 mM NO_3_^−^, *AtNRT2.1* and *AtNRT2.5* expression decreased in transgenic lines (Figure 5f,g), while 50 mM NaCl treatment increased *SsNRT2.5* expression, accompanied by enhanced fresh weight, root length, and leaf NO_3_^−^ content (Figure 5). Notably, NO_3_^−^ content in transgenic lines SsP6, SsP7 and SsP11 did not decrease compared to WT under 0 mM NaCl (Figure 5), contrasting with 35S promoter driven transgenic lines, whose leaf NO_3_^−^ levels significantly declined under 50 mM NaCl (Figure 3). This suggests the *SsNRT2.5* promoter is critical for gene expression and NO_3_^−^ uptake under salinity, thus enhancing salt tolerance.

Transient transformation assays in *S. salsa* and *N. benthamiana* showed GUS staining intensity increased after salt treatment, with ProSsNRT2.5 lines exhibiting more pronounced staining (Figure 7f). In transgenic *Arabidopsis*, ProSsNRT2.5 lines showed deeper leaf staining than ProAtNRT2.5 lines, and both intensified after salt treatment, indicating salt‐stress *cis*‐elements in ProSsNRT2.5 enhance gene expression (Figure 7f). Both *SsNRT2.5* and *AtNRT2.5* transgenic lines showed increased NO_3_^−^ content, with *SsNRT2.5* transgenic lines accumulating significantly more leaf NO_3_^−^ under salinity (Figure 7b), demonstrating higher salt tolerance. These findings indicate that the pSsNRT2.5::*SsNRT2.5* transgenic lines maintain higher NO_3_^−^ content and better growth under salt stress, contributing to enhanced salt resistance.

Crop genetic improvement represents a cost‐effective strategy to address challenges in N fertiliser use for sustainable agriculture, aiming to reduce N demand while maintaining high yields. When *SsNRT2.5* combined with its promoter was introduced into rice, *SsNRT2.5* transgenic plants exhibited significantly higher germination percentage, root lengths, biomass, and NO_3_^−^ content under 50 and 100 mM NaCl treatments compared to WT (Figures 9 and 10), with reduced Na^+^ content and increased K^+^ content (Figure 10), similar to the results observed in transgenic *Arabidopsis*. Under salinity, transgenic rice showed enhanced salt tolerance, increased NO_3_^−^ content, and improved seed yield (Figures 10 and S8). Field trials have shown that overexpression of *OsNRT2.3b* increases grain yield and N use efficiency by 40% (Fan et al. 2016), while transgenic *SsNRT2.5* rice in this study showed about 30% seed weight increase compared to WT under saline conditions (Figure S8). Transgenic wheat overexpressing *NRT2.5* improves nitrogen absorption, suggesting that enhancing N absorption in *GmTDN1* transgenic wheat may contribute to drought tolerance (Zhou et al. 2022). In maize, *ZmNRT2.5* is identified to facilitate NO_3_^−^ translocation from husk leaves to kernels under low‐nitrogen conditions, which contributes to the accumulation of seed protein content. This finding thus offers a direct target for enhancing maize seed protein content via molecular‐assisted breeding (Wang et al. 2025). These findings underscore the significant value of *SsNRT2.5* for crop improvement, offering a strategy to reduce N fertiliser application while sustaining food production. Our results not only reveal novel biological functions of *SsNRT2.5* and its promoter but also provide a theoretical framework for enhancing N use efficiency (NUE) in crops. Notably, the salt‐inducible trait of its promoter is particularly valuable for maintaining NUE under salinity stress, a trait with important implications for safeguarding grain yield and reducing environmental pollution.

NO_3_^−^–N plays both nutritional and important osmotic roles in the halophytes *Suaeda* *physophora* (Song et al. 2006) and *S. salsa* (Song, Chen, et al. 2009). In halophytes, Na^+^ accumulating reduces cellular osmotic potential to maintain water uptake, while excessive Na^+^ cauces ion toxicity. In contrast, the storage and utilisation of NO_3_^−^ mitigate osmotic stress (Song et al. 2006). NO_3_^−^ not only acts as an osmotic regulator to lower cell water potential but also modulates Na^+^ distribution by regulating ion transporters such as HKT1. In rice, NO_3_^−^ induces *OsMADS27* expression, which subsequently activates OsHKT1.1. Upregulating *OsHKT1.1* enhances Na^+^ efflux or compartmentalization, reducing Na^+^ accumulation in photosynthetic tissues while promoting K^+^ accumulation to decrease osmotic potential. In this process, NO_3_^−^ acts as a signalling molecule to initiate HKT1‐mediated Na^+^ homeostasis regulation, synergizing with its own osmotic adjustment function to enhance plant salt tolerance (Alfatih et al. 2022). This mechanism indicates that NO_3_^−^ may regulate HKT1‐mediated Na^+^ efflux or compartmentalization via signalling pathways, thereby improving plant salt tolerance. Additionally, in *Arabidopsis*, NO_3_^−^ supply synergizes with Na^+^ to reduce osmotic potential (Álvarez‐Aragón and Rodríguez‐Navarro 2017). Therefore, the reason that *Arabidopsis* and rice transferring *SsNRT2.5* and its promoter maintain lower Na^+^ content may be: (1) higher NO_3_^−^ uptake in transgenic plants have higher biomass, thus leaf Na^+^ can be diluted; (2) higher levels of the expression of *HKT1* due to the role of NO_3_^−^ signal, to improve the ability of Na^+^ exclusion in the roots. As a result, salt tolerance in transgenic plants can be increased accompanying with the osmotic roles of NO_3_^−^, which can be obtained the evidence of higher biomass and lower leaf MDA content in transgenic plants compared to WT and 35S::*SsNRT2*.*5* transgenic lines.

In conclusion, genetic engineering of *SsNRT2.5* in crops offers a viable strategy for saline agriculture, reconciling NUE with salt adaptation. *SsNRT2.5* and its promoter contribute to the enhanced NO_3_^−^ absorption under salinity and low NO_3_^−^–N conditions, which can reduce N input and aligns with global efforts to mitigate fertiliser pollution, thus underscoring its dual role in promoting crop productivity and environmental sustainability.

## 4 Materials and Methods

### 4.1 Plant Materials

Seedlings from brown seeds of *S. salsa* collected from inland saline soils (N37°20′ E118°36′) in the Yellow River Delta in Shandong Province were used for *SsNRT2.5* gene and promoter cloning and expression analyses. *Arabidopsis thaliana* ecotype Columbia‐0 (Col‐0) was used as the wild‐type (WT) control, and the construct was used for *AtNRT2.5* gene and promoter cloning and expression analyses. Rice (Nipponbare genetic background) transferred *SsNRT2.5* and its promoter were used to test the function of the gene and its promoter in the crop under high salt and low NO_3_^−^ conditions.

### 4.2 Plant Growth Conditions and Treatment

The brown seeds of *S. salsa* were sown in 15 × 19 cm plastic pots filled with clean sand under a 14‐h 28°C/10‐h 25°C light/dark photoperiod in a growth chamber. There were 15 seedlings in each pot. Plants were watered with 0.5 mM NO_3_^−^ nutrient solution every day. The 0.5 mM NO_3_^−^ nutrient solution contained 0.25 mM Ca(NO_3_)_2_, 2.75 mM CaCl_2_, 2 mM K_2_SO_4_, 2 mM MgSO_4_, 1 mM KH_2_PO_4_, 45 μM Fe‐EDTA, 23 μM H_3_BO_3_, 4.55 μM MnCl_2_, 0.16 μM CuSO_4_, 0.38 μM ZnSO_4_ and 0.28 μM (NH_4_)_6_Mo_7_O_24_. After 28 days treatment, the seedlings in four pots were used for DNA and RNA extraction.

In order to assess the trend of *SsNRT2.5* expression at different time intervals, seedlings of *S. salsa* were grown hydroponically in 0.5 mM NO_3_^−^ nutrient solution with aeration by an aeration pump and the culture medium was changed once a week when they were growing true leaves. After 28 days, the seedlings were cultured with 0 mM NO_3_^−^ nutrient solution for 7 days. The 0 mM NO_3_^−^ nutrient solution contained 3 mM CaCl_2_, 2 mM K_2_SO_4_, 2 mM MgSO_4_, 1 mM KH_2_PO_4_, 45 μM Fe‐EDTA, 23 μM H_3_BO_3_, 4.55 μM MnCl_2_, 0.16 μM CuSO_4_, 0.38 μM ZnSO_4_ and 0.28 μM (NH_4_)_6_Mo_7_O_24_. After the seedlings were cultured with 0 mM NO_3_^−^ for 5 days, they were treated with 0 and 300 mM NaCl, prepared with the 0.5 mM NO_3_^−^ solution as described above. In order to avoid osmotic shock, the concentration of NaCl applied was gradually increased by 50 mM/d in the initial 4 days and 100 mM/d in the last 1 day. The root and leaf samples were collected after the highest concentration of NaCl was obtained for 0 h, 6 h, 12 h, 24 h, 2 days, 3 days and 7 days. The root samples were used for *SsNRT2.5* expression detection. All treatments were done in triplicate.

Seedlings of *Arabidopsis* were grown under a 16‐h 22°C/8‐h 18°C light/dark cycle (relative humidity was 70%, light intensity during the day period was 300 ± 50 μmol m^−2^ s^−1^) conditions. The seeds of different *Arabidopsis* lines were sown on 1/2 MS and nitrogen‐free 1/2 MS medium with 0, 0.1, 0.5 or 1 mM KNO_3_ as a nitrogen source and with 0, 50, 100 or 150 mM NaCl added to the medium. The pH was adjusted to 5.8 with KOH. Agar (9 g/L) was added before autoclaving. The physiological indices, such as root length and seedling fresh weight, were determined 10 days after the seeds were sown on the media. All treatments were done in triplicate.

The seeds of different *Arabidopsis* lines were sown into hydroponic box, with nine seedlings per box. The seedlings were cultured with 1/2 Hoagland nutrient solution, and after 14 days, the plants were treated with 0 mM NO_3_^−^ nutrient solution for 3 days and cultured with 0.5 mM NO_3_^−^ nutrient solution with 0 or 50 mM NaCl added. These seedlings were ventilated using an aeration pump, and the culture solution was replaced weekly. To avoid osmotic shock, the concentration of NaCl was gradually increased by 25 mM/d, until 50 mM. The NO_3_^−^, Na^+^ and K^+^ concentrations were determined after 50 mM NaCl was maintained for 1 day. Meanwhile, the different *Arabidopsis* lines were grown on vermiculite and nutrient soil, with 0, 50 or 100 mM NaCl added to the medium. The physiological indices including seedling fresh weight, dry weight and leaf NO_3_^−^, Na^+^ and K^+^ concentrations were determined after 100 mM was maintained for 7 days. The plant height, fresh and dry weight were determined after the plant has developed mature fruit pods (cultivation duration was 60 days). All treatments were done in triplicate.

### 4.3 Total RNA Extraction and cDNA Synthesis and RT‐qPCR

Total RNA was extracted from the *S. salsa* , *Arabidopsis* and rice roots using WARYONG Ultra Fast PlantRNA Extraction Kit according to the manufacturer's protocols. The cDNA was synthesised using the SMARTer RACE 5′/3′ Kit, 3′‐Full RACE Core Set with PrimeScript Rtase, and PrimeScript RT reagent Kit with gDNA Eraser (Takara, China) according to the manufacturer's instructions. Then RT‐qPCR assays were performed with the SYBR Green Pro Taq HS qPCR Kit using Light Cycler 96. The expression of *ACTIN* in *S. salsa* , *Arabidopsis* and rice were used for normalising respective gene expression. The relative expression of *NRT2.5* in different species were calculated using the 2^−ΔΔCt^ method. All the primers used in the RT‐qPCR analysis were listed in Table S3.

### 4.4 Cloning and Sequencing of *SsNRT2.5* and *AtNRT2.5* Genes and Their Promoter

The intermediate fragment of the *SsNRT2.5* gene was obtained according to the known sequences retrieved from the RNA sequencing (RNA‐seq) of *S. salsa* (Table S1). The *S. salsa* cDNA was used as a template, and primers were designed to obtain a 1212 bp intermediate fragment, and then specific primers were designed from the intermediate fragment for 5′ and 3′ rapid amplification of cDNA ends (RACE) to obtain the full‐length *SsNRT2.5* coding sequence. The promoter sequence of the *SsNRT2.5* was obtained using the Genome Walking Kit (code 6108) as a template and the known sequence of the *SsNRT2.5* as a template, and the flanking sequence of the 5′ end of the DNA was obtained using the Genome Walking Kit (code 6108). The *AtNRT2.5* gene and its promoter were retrieved from TAIR (https://www.arabidopsis.org/), and using the *Arabidopsis* cDNA and genomic DNA as templates, primers were designed to amplify the promoter and coding region (primer sequences listed in Table S3). All primers were designed using Primer Premier 5 software. Nucleic acid sequences were translated into protein sequences using BLASTP online and DNAman software for homologous sequence alignment and homology analysis. Phylogenetic relationships of the *SsNRT2.5* amino acid sequence with orthologs from other plant species were analysed using MEGA5 software.

### 4.5 Plasmid Construction and Plant Transformation

The full‐length coding sequences of *SsNRT2.5* and *AtNRT2.5*, along with their respective promoter regions, were cloned into plant binary vectors to generate the following constructs: 35S::*SsNRT2.5‐sGFP*, pSsNRT2.5::*SsNRT2.5‐sGFP*, 35S::*AtNRT2.5‐sGFP*, pAtNRT2.5::*AtNRT2.5‐sGFP*, pSsNRT2.5::*GUS* and pAtNRT2.5::*GUS*. These constructs were introduced into *Arabidopsis* via *Agrobacterium tumefaciens* (GV3101)‐mediated inflorescences‐infected transformation method (Zhang et al. 2006). The pSsNRT2.5::*SsNRT2.5‐sGFP* was transformed into rice ( *Oryza sativa* cv. Nipponbare) using the *Agrobacterium* (EHA105)‐mediated genetic transformation.

To generate CRISPR/Cas9 knockout lines of *AtNRT2.5*, the intermediate vector AtU6‐26‐sgRNA‐SK for single guide RNA (sgRNA) cassette and the final vector pCAMBIA1300‐pYAO::Cas9 for transformed plants were constructed according to Yan et al. (2015) using the YAO promoter‐driven CRISPR/Cas9 system.

For transient expression assays, 35S::*SsNRT2.5‐*sGFP, pSsNRT2.5::*SsNRT2.5‐*sGFP were co‐transfected with the empty 35S::sGFP vector into the lower epidermis of *Nicotiana benthamiana* leaves. GFP fluorescence was visualised using a two‐photon laser scanning confocal microscope (TCS SP8 MP, Leica, Germany) with a 488 nm excitation source.

Based on the identification of salt‐responsive *cis‐*acting element in the *SsNRT2.5* promoter, the promoter sequence was subjected to 5′ deletions. Full length and truncated segments (2774 bp as Q1, 1357 bp as Q2 and 645 bp as Q3 upstream of the ATG start codon) of the promoter were constructed into GUS reporter vectors (Q1/Q2/Q3::*GUS*). These constructs, along with pSsNRT2.5::*GUS*, pAtNRT2.5::*GUS* and Q1/Q2/Q3::*GUS* were used for transfecting *N. benthamiana*, *S. salsa*, and *Arabidopsis*.

### 4.6 Transient Expression Assay Mediated by *Agrobacterium*

The expression vector containing the 5′ deletion fragment was transferred into *Agrobacterium rhizogenes* EHA105 in YEP (50 mg/mL kanamycin, 25 mg/mL rifampicin, 2 μmol/L 5‐azacytidine and 20 μmol/L acetosyringone (AS)) at 28°C with shaking for overnight. After centrifugation at 6000 rpm for 10 min, the bacterial solution was adjusted until the OD600 = 0.7 with the transformation solution, and the solution was incubated at room temperature for 1 h.

For *S. salsa* transformation, the roots and apical young leaves were excised and sterilised by soaking in 50% ethanol for 30 s and 2% NaClO for 1 min. After sterilisation, the samples were rinsed three times with sterile distilled water and gently dried using sterile filter paper. Sterilised tissues were immersed in the Agrobacterium suspension, incubated on a shaker at 25°C and 90 rpm for 2 h, supplemented with fresh infiltration buffer. The transformation was continued in the shaker for 1.5 h. The material was then transferred to the washing solution. They were washed rapidly for 1 min and blotted dry with filter paper. The transiently transformed plants were directly inserted into 1/2 MS solid medium (1% sucrose, 7 g/L agar, 150 μmol/L AS and 20 mg/L DTT), and the transiently transformed plants were cultured for 36 h. Untreated *S. salsa* was used as a negative control, and *S. salsa* transfected with 35S: GUS vector was used as a positive control sprayed with 100 mM NaCl for 24 h. The material was harvested for GUS activity staining (Li et al. 2025).

For *N. benthamiana* transformation, *Agrobacterium* cultures were prepared as described above, adjusted to OD600 = 1, and infiltrated into the abaxial surface of fully expanded leaves using a syringe without a needle. Infiltrated plants were incubated in the dark for 12 h before transfer to normal growth conditions. Untransformed tobacco and plants transfected with 35S::GUS were used as negative and positive controls, respectively. Following treatment with 100 mM NaCl for 24 h, leaves were harvested for GUS activity analysis.

### 4.7 GUS Assay

Transgenic *Arabidopsis* lines harbouring the pSsNRT2.5::GUS construct were used for the experiments. Roots, leaves, flowers, fruit pods, and seeds at different growth stages were collected to detect the β‐glucuronidase (GUS) activity by incubating with GUS Stain Kit (Cat: G3061). The transformed materials were removed and immersed in GUS staining solution at 37°C for 4–12 h in the dark. GUS staining was then observed by sequential elution with anhydrous ethanol until the background colour of the materials was white. Additionally, transgenic *Arabidopsis* seeds were cultivated on 1/2 MS medium supplemented with 0, 50 or 100 mM NaCl for 7 days to observe variations in GUS staining patterns.

### 4.8 Measurement of NO_3_ ^−^, Na^+^ and K^+^ Concentration

For *S. salsa*, *Arabidopsis* and rice, 0.1 g sample of the leaves or roots was added to 1 mL of distilled water, homogenised and incubated in a thermostatic water bath at 90°C for 30 min with constant shaking and then centrifuged at 12 000 rpm for 15 min after cooling and the supernatant was taken for measurement.

The concentration of NO_3_^−^ was determined using the Plant Nitrate Nitrogen Content Assay Kit (Cat:BC1505). After the solution was filtered, the concentration of Na^+^ and K^+^ was determined using a flame photometer (Flame Photometer 410, Sherwood Scientific Ltd., Cambridge, UK). Each treatment was done in triplicate.

### 4.9 Statistical Analysis

Experimental data were processed using Excel 2019, graphs were generated using Graphpad prism 5, and all analyses of the experimental data were analysed using SAS (SAS Institute Inc., 1989) and SPSS Statistics 26 (IBM, Chicago, IL) software for factorial statistical analysis. Two‐way ANOVA with the least significant difference (LSD) test was conducted using SAS to assess significance, with *p* < 0.05 defined as statistically significant. One‐way ANOVAs were performed using SPSS Statistics 26, and treatment means were separated by Duncan's multiple range test at *p* < 0.05.

## Author Contributions

R.L., C.L. and J.S. conceived and designed the research. R.L., C.L., R.t.Z., C.S., Y.Z. and R.x.Z. performed the experiments and analysed the data. R.L. wrote the manuscript draft. N.S., L.W. and J.S. finalised the writing and revision of the manuscript.

## Funding

This work was supported by National Natural Science Foundation of China, 32171499, 32501392.

## Conflicts of Interest

The authors declare no conflicts of interest.

## Supporting information


**Figure S1:** Analysis of *SsNRT2.5* expression and NO_3_^−^ content in different seed types of *S. salsa* .

**Figure S2:** Sequencing peak maps for verifying gene‐edited sequences of *atnrt2.5* mutants (M2, M22, M76 and M15).

**Figure S3:** Phenotypic analysis of *atnrt2.5* mutants (M2, M22) *Arabidopsis* under different concentrations of NaCl and NO_3_^−^ treatments.

**Figure S4:** Phenotype analysis of *SsNRT2.5* promoter—transgenic *Arabidopsis* in 1/2 MS medium under salt stress.

**Figure S5:** Biomass and NO_3_^−^content analysis of *SsNRT2.5*‐transgenic *Arabidopsis* under salt stress.

**Figure S6:** Responses of WT and *SsNRT2.5*‐transgenic *Arabidopsis* to different NaCl concentrations.

**Figure S7:** Phenotypes and NO_3_^−^ accumulation in WT, *AtNRT2.5* overexpression lines (AtOE4, AtOE6) and *SsNRT2.5* overexpression lines (SsOE15, SsOE35) under different NaCl concentrations.

**Figure S8:** Effects of salinity on rice transferring ProSsNRT2.5::*SsNRT2.5* lines regarding seed development and related characteristics.

**Table S1:** SsNRT2.5 and AtNRT2.5 genes and their promoter.

**Table S2:** Analysis results of *cis*‐acting elements in the promoter of *Suaeda salsa SsNRT2.5* gene (ProSsNRT2.5) and *Arabidopsis thaliana* *AtNRT2.5* gene (ProAtNRT2.5).

**Table S3:** Primers used in the experiment.

## Data Availability

All data included in this study are available within the article and its Supporting Information.
