# Supplementary figures and images for: The Advantaged Salt Inducible Suaeda salsa SsNRT2.5 and Its Promoter Significantly Enhance Nitrate Transport Efficiency and Salt Tolerance in Transgenic Arabidopsis and Rice
Source: Plant Biotechnol J. 2026 Mar 10;24(6):4084–101. doi: 10.1111/pbi.70600 (PMC13205868; doi:10.1111/pbi.70600)

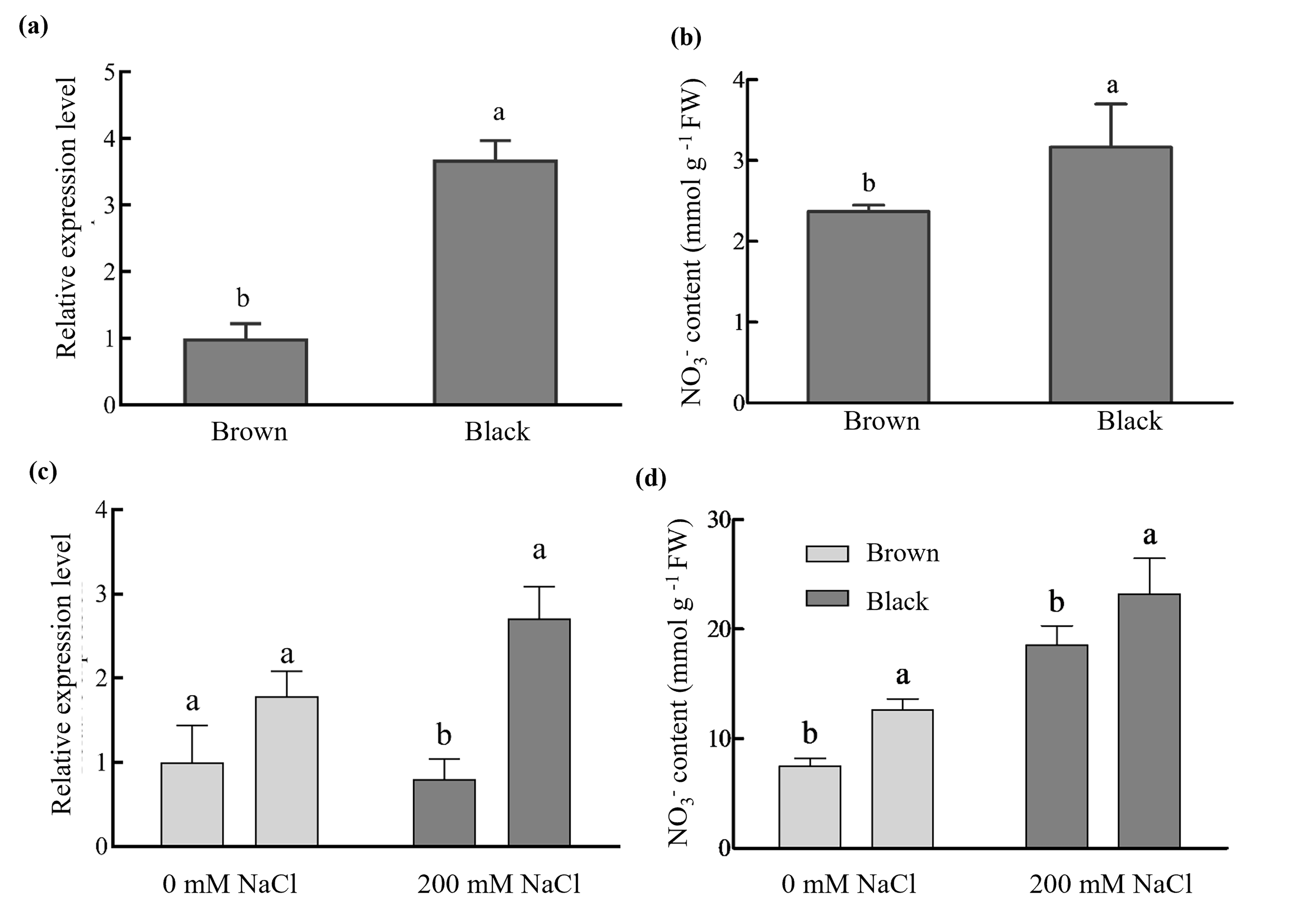

Supplement: Supplementary file 1 — Figure S1: Analysis of SsNRT2.5 expression and NO3 − content in different seed types of S. salsa . [file PBI-24-4084-s011.tif]

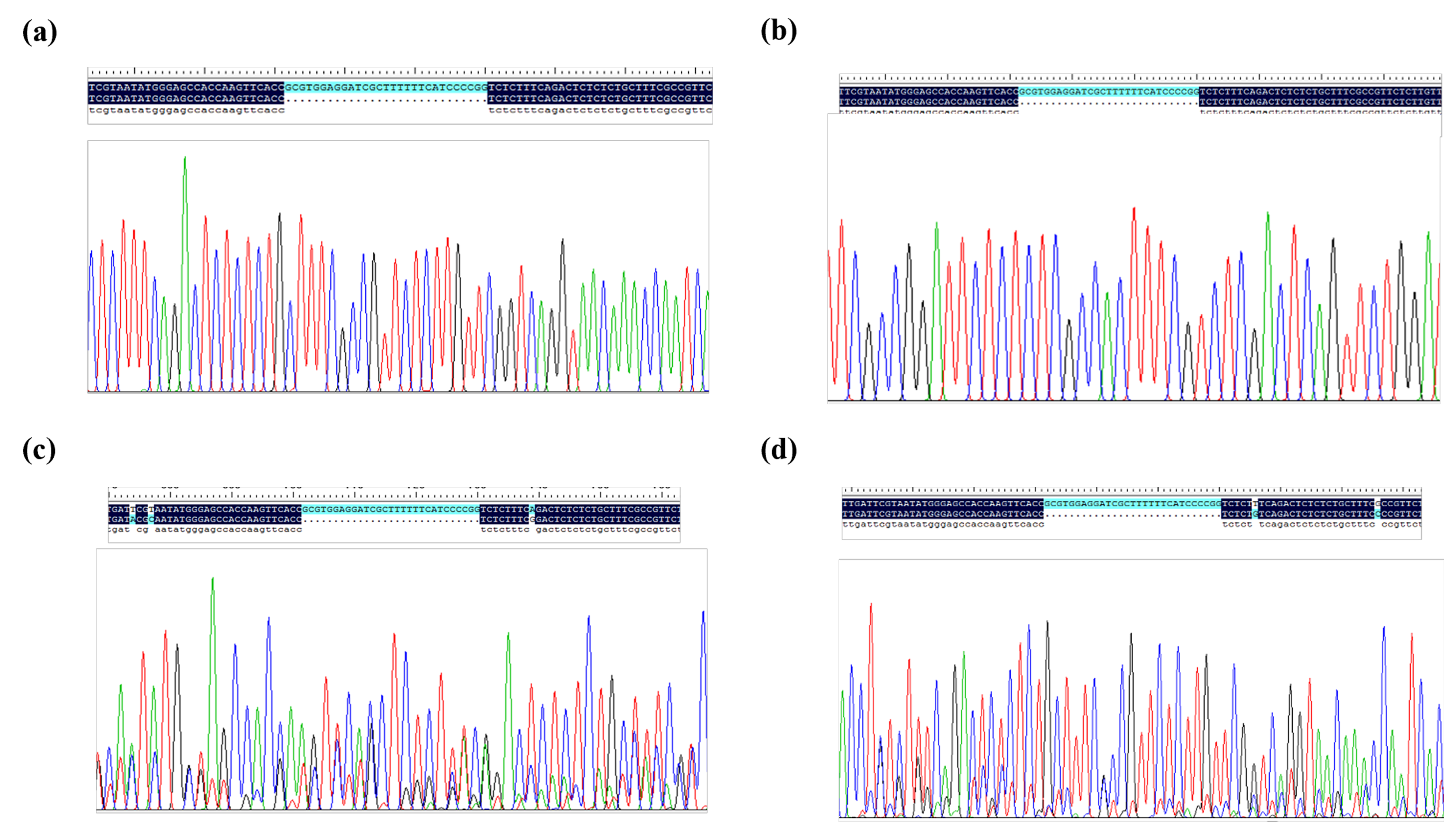

Supplement: Supplementary file 2 — Figure S2: Sequencing peak maps for verifying gene‐edited sequences of atnrt2.5 mutants (M2, M22, M76 and M15). [file PBI-24-4084-s002.tif]

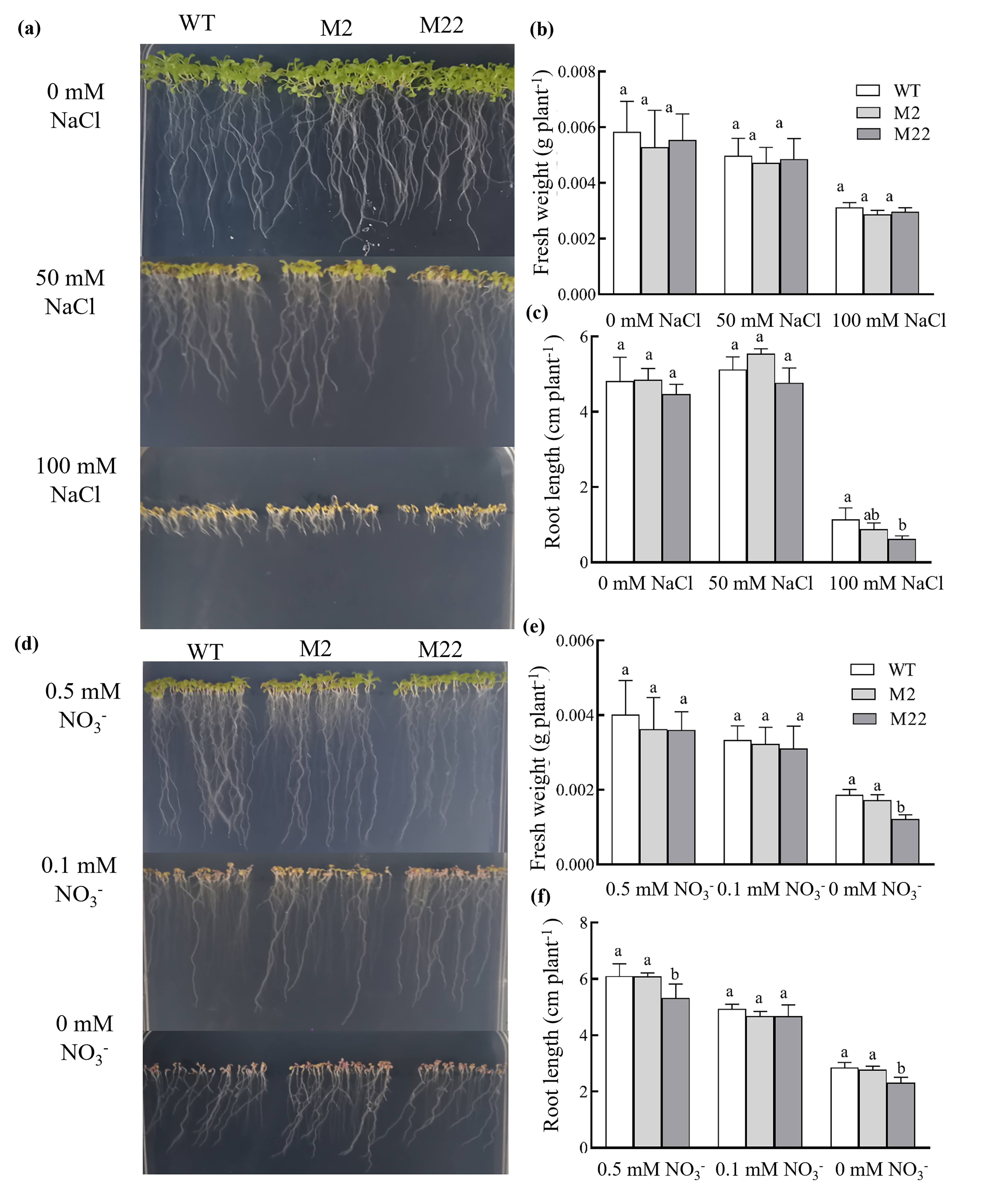

Supplement: Supplementary file 3 — Figure S3: Phenotypic analysis of atnrt2.5 mutants (M2, M22) Arabidopsis under different concentrations of NaCl and NO3 − treatments. [file PBI-24-4084-s009.tif]

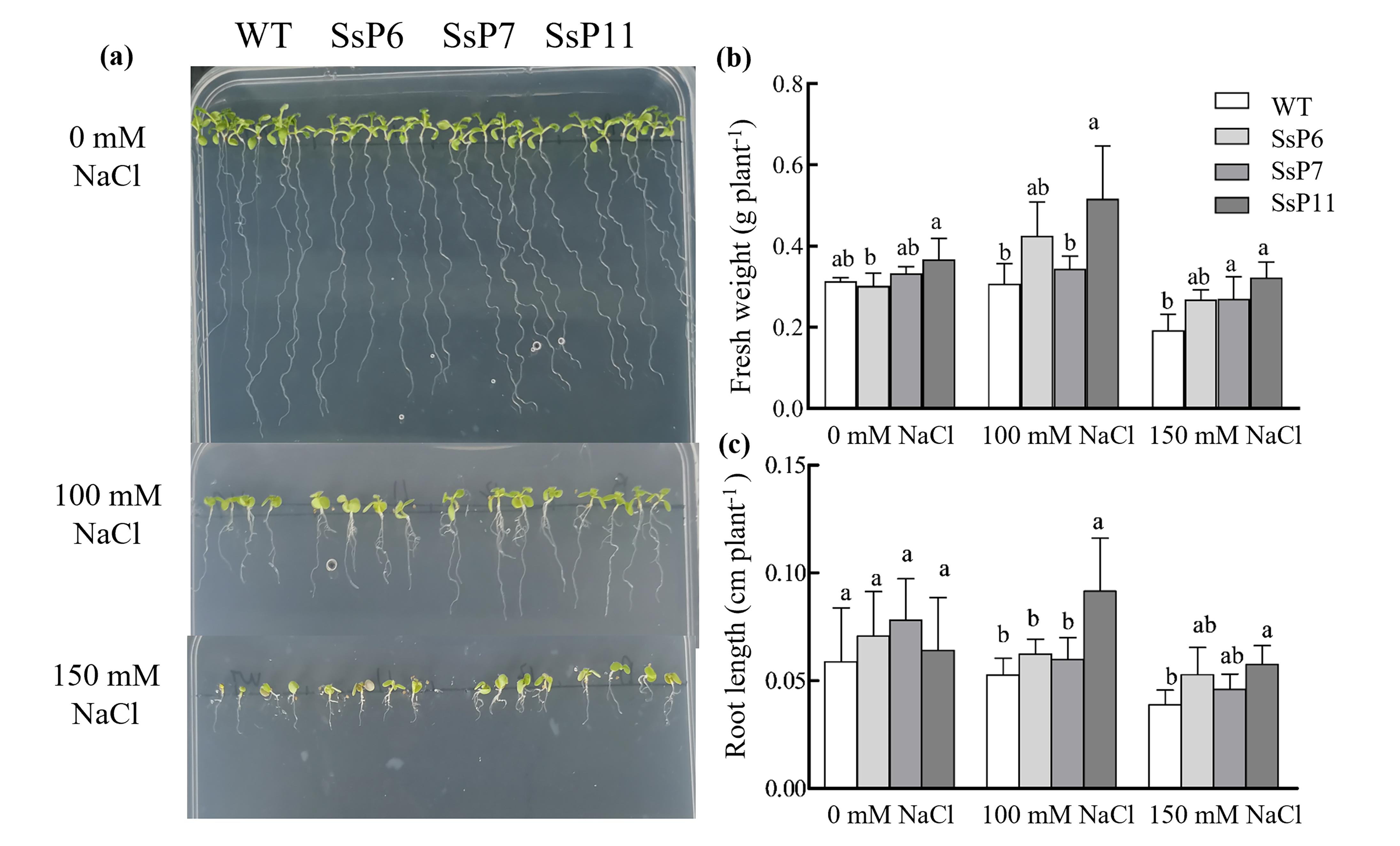

Supplement: Supplementary file 4 — Figure S4: Phenotype analysis of SsNRT2.5 promoter—transgenic Arabidopsis in 1/2 MS medium under salt stress. [file PBI-24-4084-s006.tif]

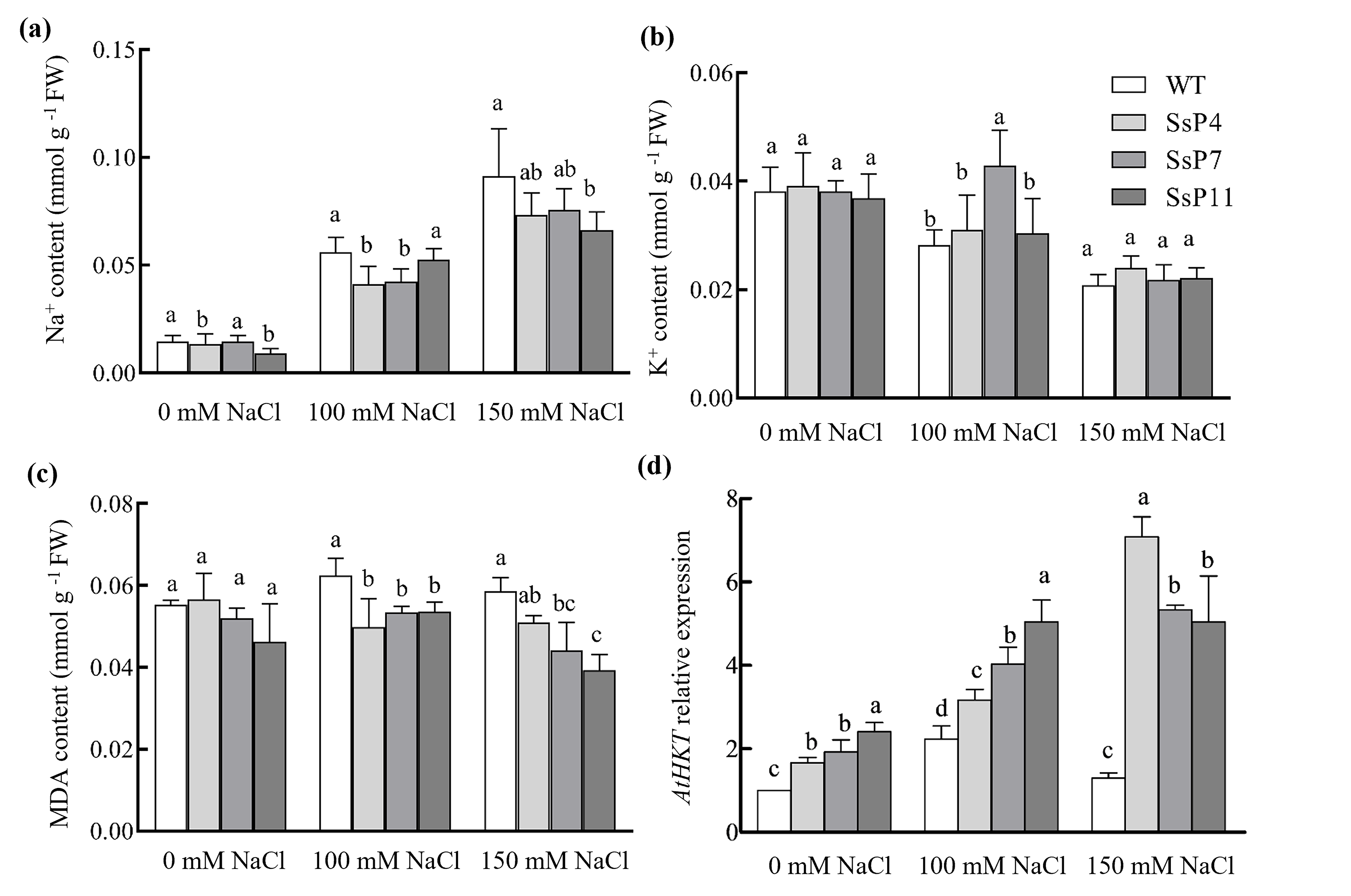

Supplement: Supplementary file 6 — Figure S6: Responses of WT and SsNRT2.5‐transgenic Arabidopsis to different NaCl concentrations. [file PBI-24-4084-s010.tif]

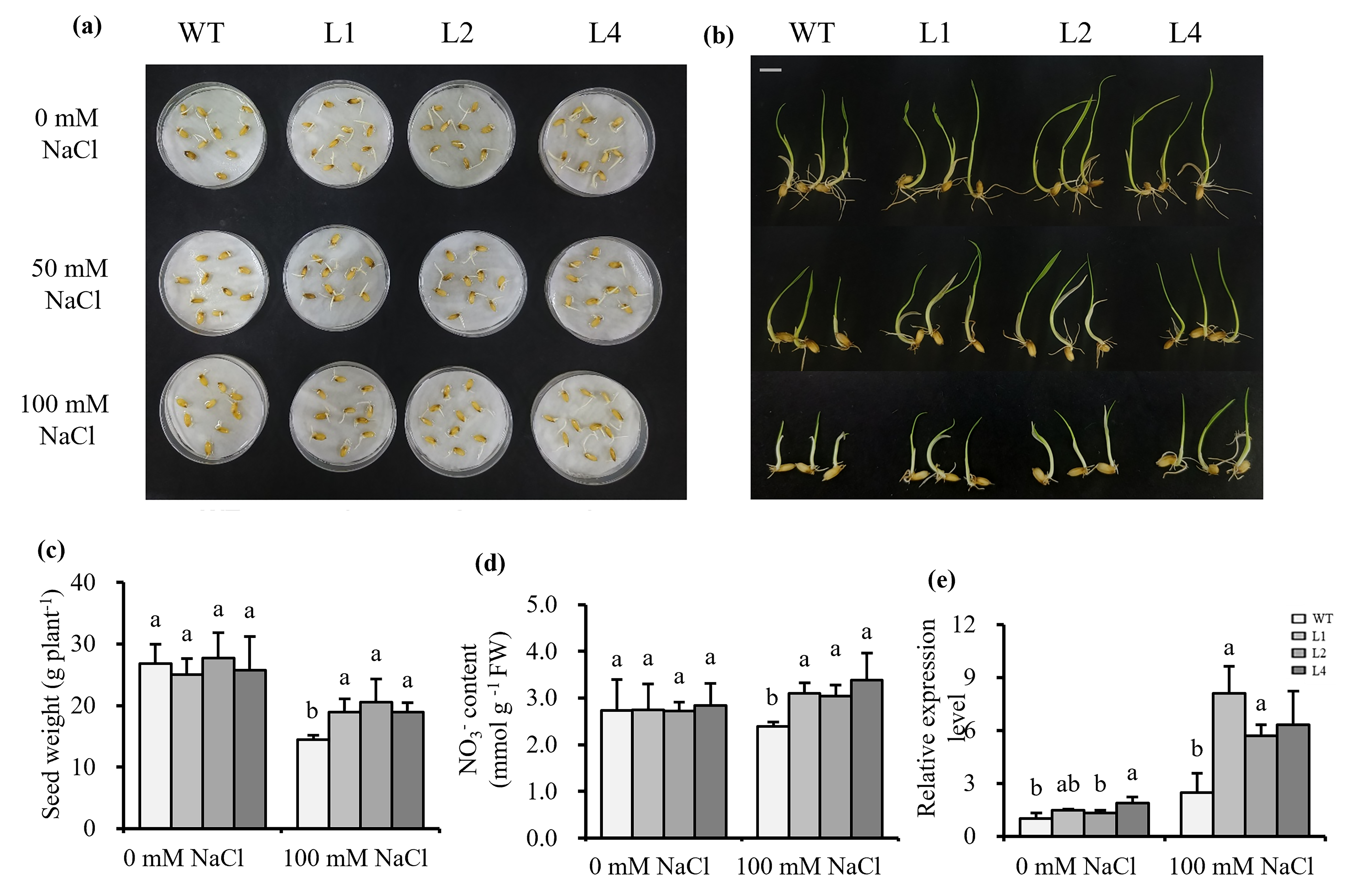

Supplement: Supplementary file 8 — Figure S8: Effects of salinity on rice transferring ProSsNRT2.5::SsNRT2.5 lines regarding seed development and related characteristics. [file PBI-24-4084-s007.tif]
