# Supplementary material for: The Advantaged Salt Inducible Suaeda salsa SsNRT2.5 and Its Promoter Significantly Enhance Nitrate Transport Efficiency and Salt Tolerance in Transgenic Arabidopsis and Rice
Source: Plant Biotechnol J. 2026 Mar 10;24(6):4084–101. doi: 10.1111/pbi.70600 (PMC13205868; doi:10.1111/pbi.70600)
Supplement: Supplementary file 9 — Table S1: SsNRT2.5 and AtNRT2.5 genes and their promoter. [file PBI-24-4084-s008.docx]

**Table S2** Primers used in the experiment.

| Name | Sequence (5'-3') | Applicaition |
| --- | --- | --- |
| *SsNRT2.*5-F1 | CAGTAGCAAAGCCTCATAT | Intermediate fragment of *SsNRT2.*5 |
| *SsNRT2.*5-R1 | TGGTTGTTATGCCTAATGT |  |
| *SsNRT2.*5-R2 | AGCAGACATGTAAACTGCAGGTAC | 5' RACE |
| *SsNRT2.*5-R3 | GTCCTGTCAAGTCACAGGCGGTAC |  |
| 3' RACE Adaptor | Contains dT area and Adaptor Primer specially designed by Takara | 3' RACE |
| 3' RACE OuterPrimer | GATAGTCCAAACACTAAGCGGTGT |  |
| 3' RACE InnerPrimer | CTCAGTACTTTTGGAGCATCGGTT |  |
| 3' RACE Control Outer Primer | GAGTGCAGGACATTGTGGTAGGG |  |
| 3' RACE Control Inner Primer | ATCTTTGACTGCCGTTCTCGACC |  |
| *SsNRT2.*5-F01 | TGCTTATCTATTACAAAACAAAAG | Full length of *SsNRT2.*5 cDNA |
| *SsNRT2.*5-R01 | TTGATTGGAGATACATACAAGAAG |  |
| pSsNRT2.5-F | ATGACATCCACAAACAACGGAGGAATG | Full length of *SsNRT2.*5 DNA |
| pSsNRT2.5-R | TTAGACATTGTTAGTTTGCAAATTATCTTCTTTGG |  |
| SP1 | GAATGTTGCGGGTGAGTTTGCTA | TAIL-PCR of promoter cloning |
| SP2 | TGACCCTATCCCAGCATTGCCAAT |  |
| SP3 | CCAACTAAGATGAAACGCCTGCAT |  |
| SP4 | TGGGAGCCATTGCAAGCAATGGGAA |  |
| SP5 | ATACTTGATTGTCCGAATCCCATATCC |  |
| SP6 | CAGATAGTTGTGCTCAGCCTTAGC |  |
| pSsNRT 2.5-YF1 | GCTCCCAATTAATACACAC | Full length of *SsNRT2.*5 promoter |
| pSsNRT 2.5-YR1 | CTGTGGCAGCAGTATTACTGAGG |  |
| pSsNRT 2.5-F1 | AGAATGGCACAAGATTGTATGTTG |  |
| pSsNRT 2.5-R1 | GATCACTCTACTTGACTTTGGGTAG |  |
| ProSsNRT2.5-F1 | ttcggtccccagaATGGCACAAGATTGTATGTTG | Vector construction (pCAMBIA1300) |
| ProSsNRT2.5-R1 | accgagctcgatcACTCTACTTGACTTTGGGTAG |  |
| *SsNRT2.5-*INF | cggtacccggggatccaTGACATCCACAAACAACGGAGGAATG | Vector construction (pCAMBIA1300) |
| *SsNRT2.5-*INR | cccttgctcaccatgtcgACGACATTGTTAGTTTGCAAATTATCTTCTTTGG |  |
| *AtNRT2.5*-F1 | ATTG AGAGAGTCTGAAAGAGACCG | Knockout of *Arabidopsis* homozygous mutants |
| *AtNRT2.5*-R1 | AAAC CGGTCTCTTTCAGACTCTCT |  |
| *AtNRT2.5*-F2 | ATTG AGCCACCAAGTTCACCGCG |  |
| *AtNRT2.5*-R2 | AAAC CGCGGTGAACTTGGTGGCT |  |
| Pro-F: | cctctagagtcgacctgcagAGAATGGCACAAGATTGTATGTTG | promoter vector construction (pCAMBIA3301) |
| Pro-R: | taccctcagatctaccatggGATCACTCTACTTGACTTTGGGTAG | 5' deletion promoter vector construction (pCAMBIA3301) |
| Pro-1357-F | cctctagagtcgacctgcagCTGTGATAGGTTGGCACAATC |  |
| Pro-645-F | cctctagagtcgacctgcagCTGCAGACAAAAGTTTTGGTATTACC |  |
| *SsActin*-F | GCTCTACCCCATGCAATCCT | RT-qPCR analysis |
| *SsActin*-R | TGCTCTTGGCAGTCTCTGATT |  |
| *SsNRT2.*5-F | CTTCTAAGGTGTTATATCA |  |
| *SsNRT2.*5-R | TAAGTAGTAAGCAATGAC |  |
| *Atactin*-F | TGGTCGTACCACAGGTATTGTTGTT |  |
| *Atactin*-R | AAGGTCGAGACGAAGGATAGCAT |  |
| *AtNRT2.5*-F | GATCTTTTCGGTCCACGTCTAG |  |
| *AtNRT2.5-*R | AATCTCACCATGATAAACCCGA |  |
| *AtSOS1*-F | ATTTTGATGCAGTCAGTGGATG |  |
| *AtSOS1*-R | GCAAGCAGATTCTAGTCTTTCG |  |
| *AtHKT1*-F | CCTCTACGTCTCCTATTTCACC |  |
| *AtHKT1*-R | ACTAAGAACCACCGAGTACAAG |  |
| *AtNHX1*-F | GTTGCCCTTATGATGCTTATGG |  |
| *OsActin*-F | ACCTTGCTGGGCGTGAT |  |
| *OsActin*-R | AGGGCGATGTAGGAAAGC |  |
| *OsNRT2.3(2.5)* -F | ACGGCACAAAGTACAAGACG |  |
| *OsNRT2.3(2.5)* -R | CCACTGCGGGAAGTAGATG |  |
