# Supplementary material for: The Advantaged Salt Inducible Suaeda salsa SsNRT2.5 and Its Promoter Significantly Enhance Nitrate Transport Efficiency and Salt Tolerance in Transgenic Arabidopsis and Rice
Source: Plant Biotechnol J. 2026 Mar 10;24(6):4084–101. doi: 10.1111/pbi.70600 (PMC13205868; doi:10.1111/pbi.70600)
Supplement: Supplementary file 11 — Table S3: Primers used in the experiment. [file PBI-24-4084-s003.docx]

**Table S2** Analysis results of *cis*-acting elements in the promoter of Suaeda salsa *SsNRT2.5* gene (ProSsNRT2.5) and Arabidopsis thaliana *AtNRT2.5* gene (ProAtNRT2.5)

| Element Name | Hit Sequence | Element Function | ProSsNRT2.5 | ProAtNRT2.5 |
| --- | --- | --- | --- | --- |
| AGTCA-element | AGTCA  /ACTCA | Core element for nitrate induction | 8 | 3 |
| GATABOX | GATA | Nitrate induction element | 13 | 8 |
| NPR | CCGTTTTG | Nitrate induction element | 3 | 1 |
| GT-1 | GAAAAA | Salt stress response element | 5 | 3 |
| DE Motif | CGAACTT | Nitrogen-related metabolic element | 1 | 0 |
| TATABOX5 | TTATTT | Nitrogen metabolism regulatory element | 10 | 6 |
| MYBR | TGGTTAG | Element responsive to drought, high salt, and low temperature | 3 | 1 |
| DRE | CCGA | Element responsive to dehydration, high salt, and low temperature | 6 | 1 |
| ABRE | ACGTG | Abscisic acid response element | 2 | 1 |
| AAGAA-motif | GAAAGAA | Abscisic acid-related | 1 | 0 |
| ARE | AAACCA | Anaerobic induction | 5 | 3 |
| TGACG-motif | TGACG | *Cis*-acting element involved in MEJA response | 8 | 3 |
| G-BOX | AACGTG | MeJA response | 1 | 0 |
| MBS | CAACTG | MYB binding site, involved in drought induction | 3 | 1 |
| MYB | TAACCA, WAACCA, TAACAAA | Related to abscisic acid and gibberellin | 5 | 3 |
| MYC/ MYCATERD1 | CANNTG | NAC binding motif, related to abscisic acid and drought | 1 | 0 |
| SARE | TTCGACCATCTT | *Cis*-acting element for salicylic acid response | 1 | 0 |
| RY-element | CATGCATG | Seed-specific regulation | 1 | 0 |
